# Supplementary material for: Commitment and oncogene-induced plasticity of human stem cell-derived pancreatic acinar and ductal organoids
Source: Cell Stem Cell. 2021 Jun 3;28(6):1090–1104.e6. doi: 10.1016/j.stem.2021.03.022 (PMC8202734; doi:10.1016/j.stem.2021.03.022)
Supplement: Document S1. Figures S1–S7 [file mmc1.pdf]

Cell Stem Cell, Volume *28*

Supplemental Information

Commitment and oncogene-induced plasticity of human stem cell-derived pancreatic acinar and ductal organoids

Ling Huang, Ridhdhi Desai, Daniel N. Conrad, Nayara C. Leite, Dipikaa Akshinthala, Christine Maria Lim, Raul Gonzalez, Lakshmi B. Muthuswamy, Zev Gartner, and Senthil K. Muthuswamy

A

	iPS11	iPS13
**Donor Age**	26	27
**Donor Gender**	Male	Female
**Donor weight (lbs)**	215	106
**Donor BMI (CDC calculator)**	32.7	21.4
**Donor Race**		
**Health Information**	Healthy	Healthy
**Tissues used for generating iPS cells**	Eritroblasts	Eritroblasts
**Passages used for experiments**	9	18

B

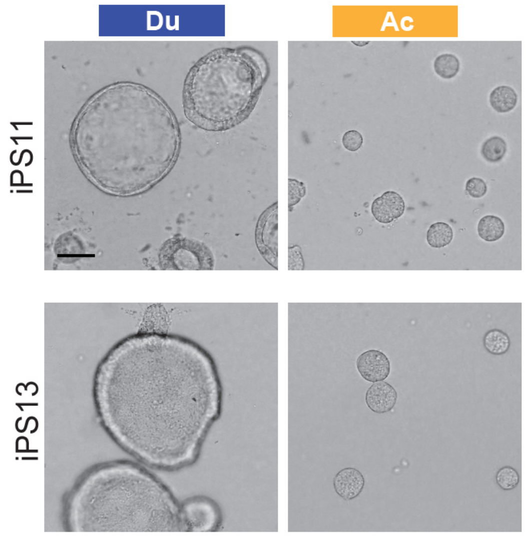

C

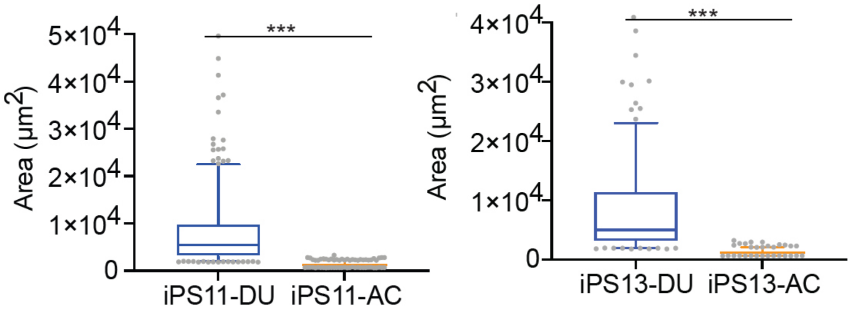

Figure S1. Induction of duct-like and acini-like organoids using progenitors derived from iPS cells (related to Figure 1) **(A)** Donor information for iPS cell lines used. **(B)** Phase contrast images of duct-like (DU, blue) and acini-like (AC, orange) organoids using iPSC-derived pancreatic progenitors. Scale bard, 50 μm. **(C)** Quantification of organoid sizes (N = minimal 200). Whisker-box plot, range 5-95%; center lines indicate median values; grey dots represent individual measurements. ***, p<0.001. Results were summary from three independent cultures.

A

	Gene Expression (arbitary unit, x10-3)		
	PP	DU	AC
PDX1	29.72	78.85	106.52
SOX9	25.97	229.48	4.66
HNF1B	141.21	175.75	84.28
HNF1A	21.85	57.07	15.53
RBPJL	4.12	12.68	12.20
RBPJ	630.67	1742.17	639.18
CPA2	2.62	6.34	4.66
CEL	6.12	6.34	15.31
PNLIP	1.75	0.00	18.42
CTRB1	0.00	0.00	3.88
CTRC	2.62	0.00	4.66

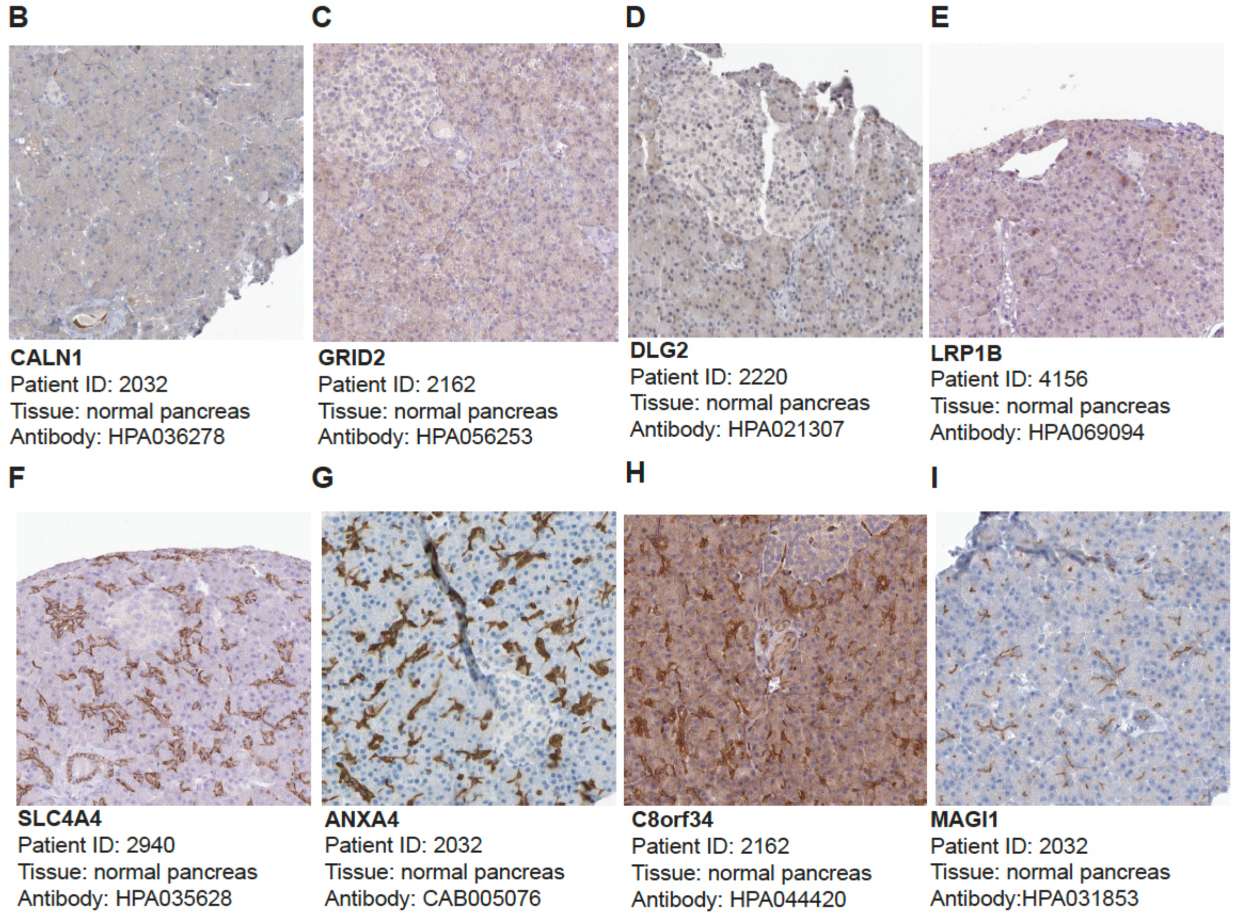

Figure S2. Single nuclei RNA sequencing detects expression of pancreatic differentiation markers in organoids (related to Figure 2) **(A)** Expression of classical pancreatic lineage markers in different cell groups. Results were obtained from two independent organoid cultures and separate sequencing. **(B-E)** Expression of acini-like organoid-enriched markers in normal human pancreas. Images obtained from Human Protein Atlas. **(F-I)** Expression of duct-like organoid-enriched markers in normal human pancreas. Images obtained from Human Protein Atlas.

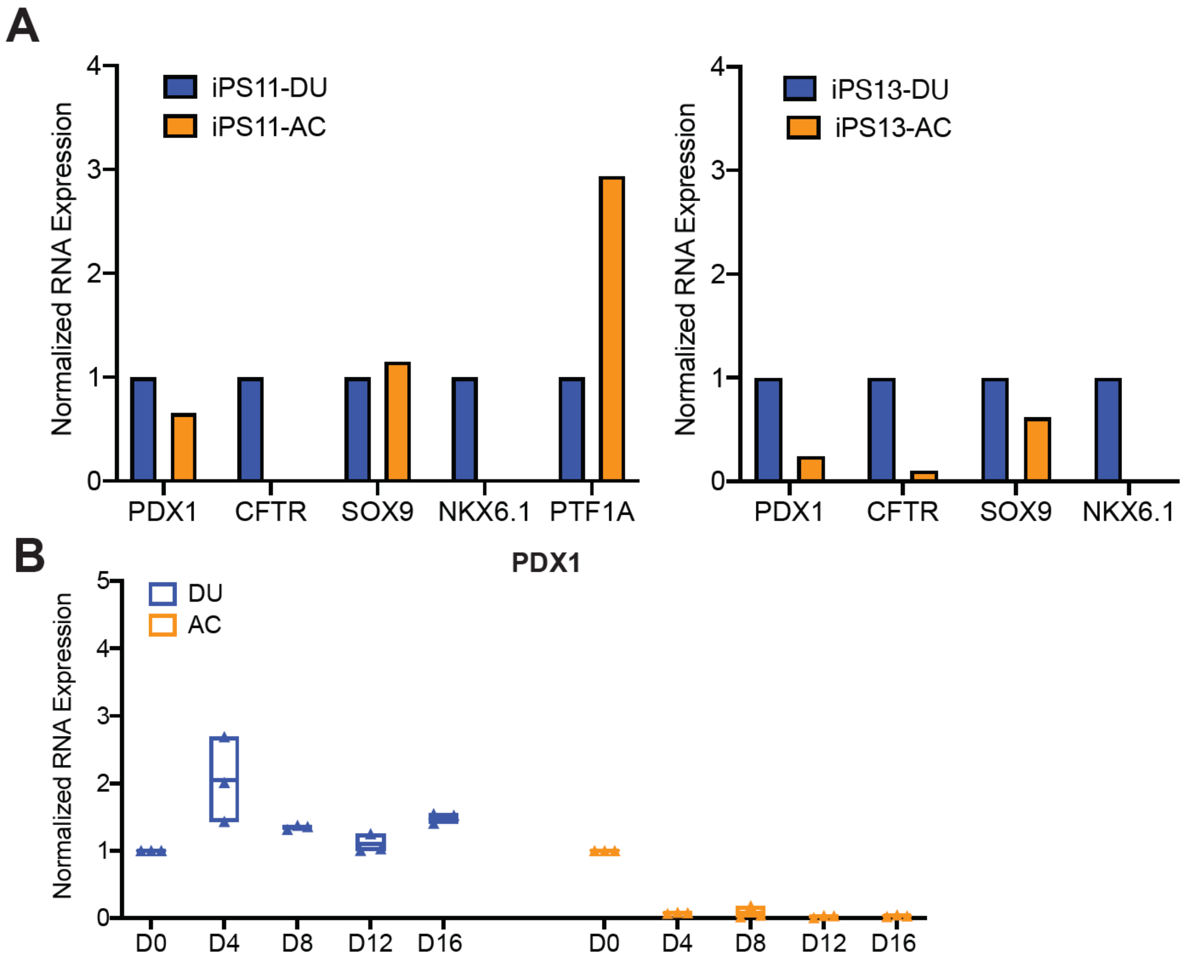

Figure S3. Expression of Pancreatic lineage markers during organoid morphogenesis (related to Figure 3) (A) Pancreatic lineage marker expression in organoids induced from iPSC-derived pancreatic progenitors. Results from one batch of organoid culture. **(B)** *PDX1* RNA levels were quantitated by quantitative PCR. Floating column charts represent RNA measurements from quantitative PCR (N=3, independent cultures); Hinges represent maximal and minimal values, central lines indicate mean values; dots represent individual measurements.

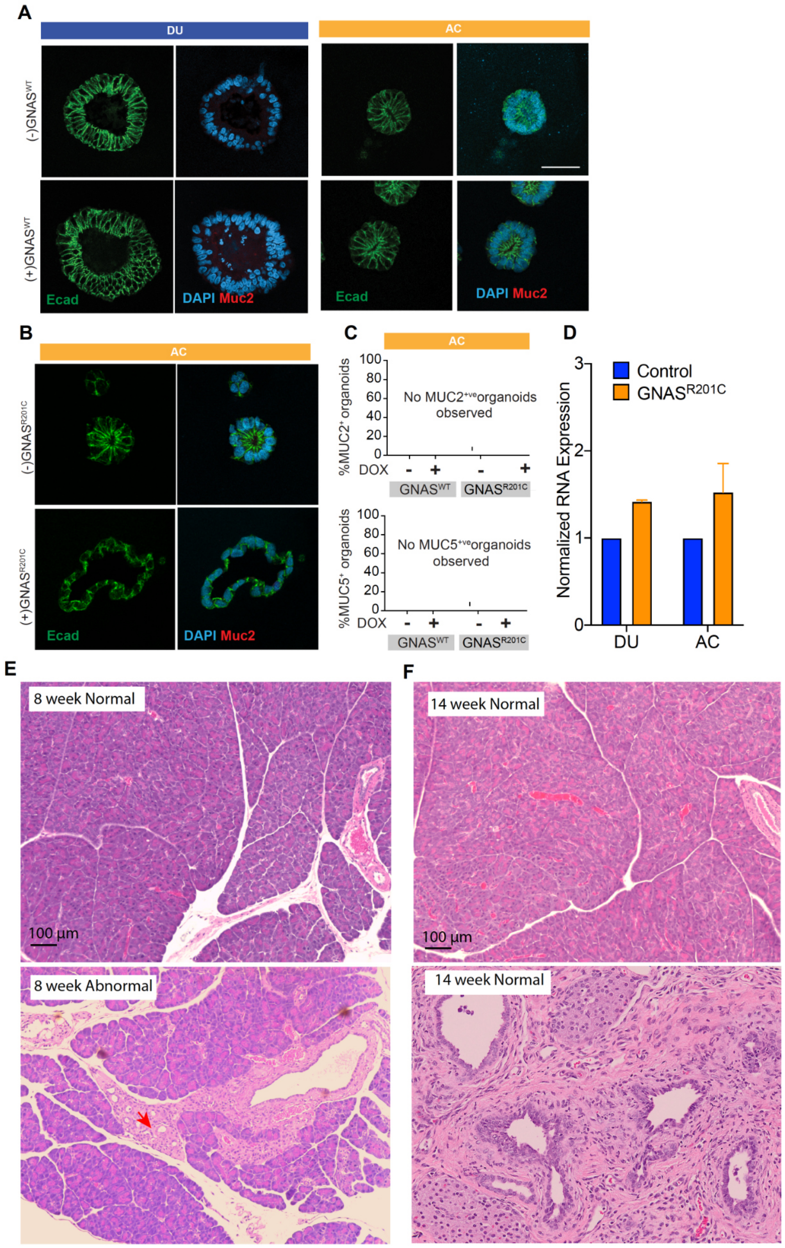

Figure S4. Effects of wildtype and mutant GNAS on organoid differentiation (related to Figure 4) **(A)** Expression of MUC2 and E-cadherin in duct-like and acini-like organoids with and without expressing wildtype GNAS (N=3, independent cultures). MUC2, red; E-cadherin, green; DAPI, blue. Scale bar, 100 µm. **(B)** Expression of MUC2 and E-cadherin in acini-like organoids with and without expressing $GNAS^{R201C}$ (N=3). MUC2, red; E-cadherin, green; DAPI, blue. Scale bar, 100 µm. **(C)** Quantification of MUC2 and MUC5AC expression in acini-like organoids with and without expressing $GNAS^{R201C}$ (N=3). **(D)** CFTR RNA expression in organoids with $GNAS^{R201C}$. N= 2, biological repeats. **(E-F)** Normal (top panels) and abnormal (bottom panels) mouse pancreatic tissues with (hematoxylin and eosin stained) at eight weeks (D) (N=8) and 14 weeks (E) (N=4) post transplantation with duct-like organoids expressing $GNAS^{R201C}$. Scale bars, 100 µm.

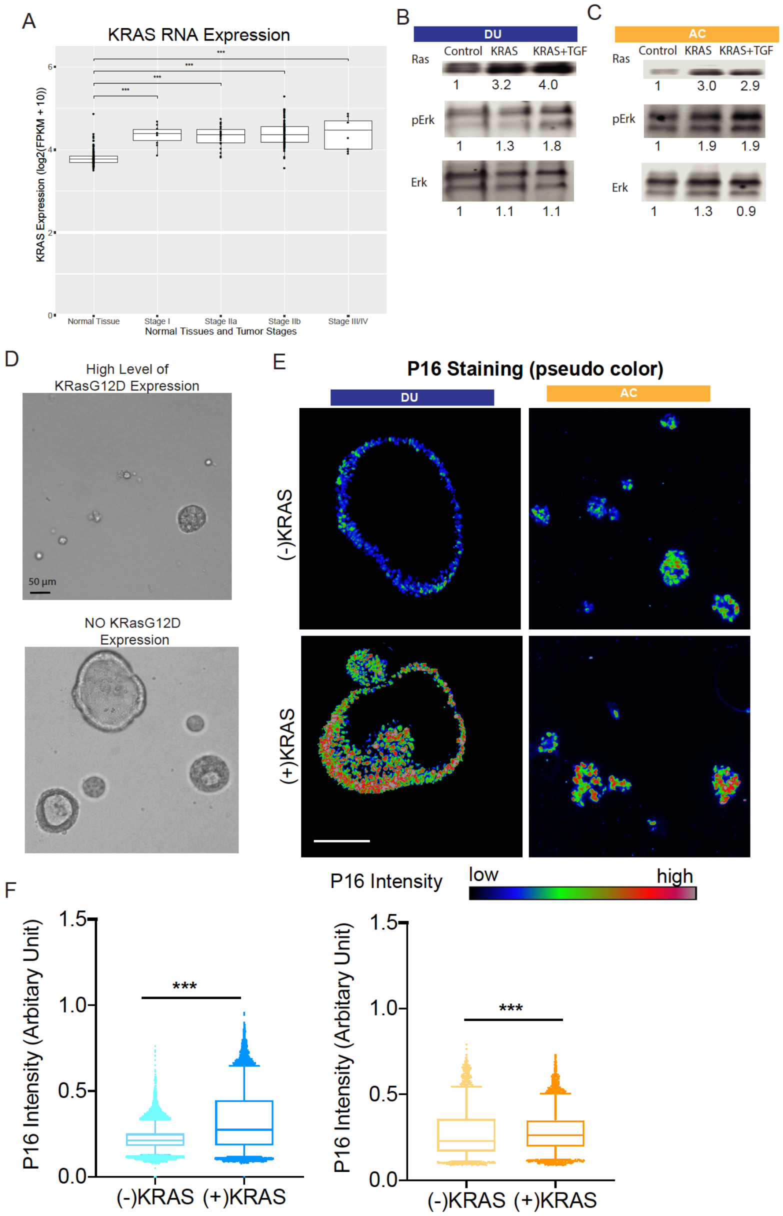

Figure S5. Effects of *KRAS*^{G12D} expression on organoids (related to Figure 5) (A) Expression of *KRAS* in the pancreas of healthy subjects and PDAC patients. Data obtained from The Cancer Genome Atlas. **(B-C)** Immunoblot analysis for KRAS and ERK phosphorylation in duct-like (B) and acini-like (C) organoids. Numbers blots represent normalized intensities of protein bands. **(D)** Phase contrast images of duct-like organoids expressing high levels of *KRAS*^{G12D} (top panel) and organoids without *KRAS*^{G12D} expression (bottom panel). **(E)** Pseudocolor images indicating p16^{INK4A} intensities in organoids without (top panel) and with (bottom panel) *KRAS*^{G12D} expression. Images converted from immunofluorescent staining of p16^{INK4A}. Scale bars, 100 µm. (**(F)** Quantification of p16^{INK4A} expression per nuclei in organoids without and with *KRAS*^{G12D} expression (N>2500, from three independent cultures). Y-axis present signals normalized to maximal values of 8 bit image (255). Whisker-box plot, range 5-95%; center lines indicate median values; grey dots represent individual measurements. ***, p<0.001.

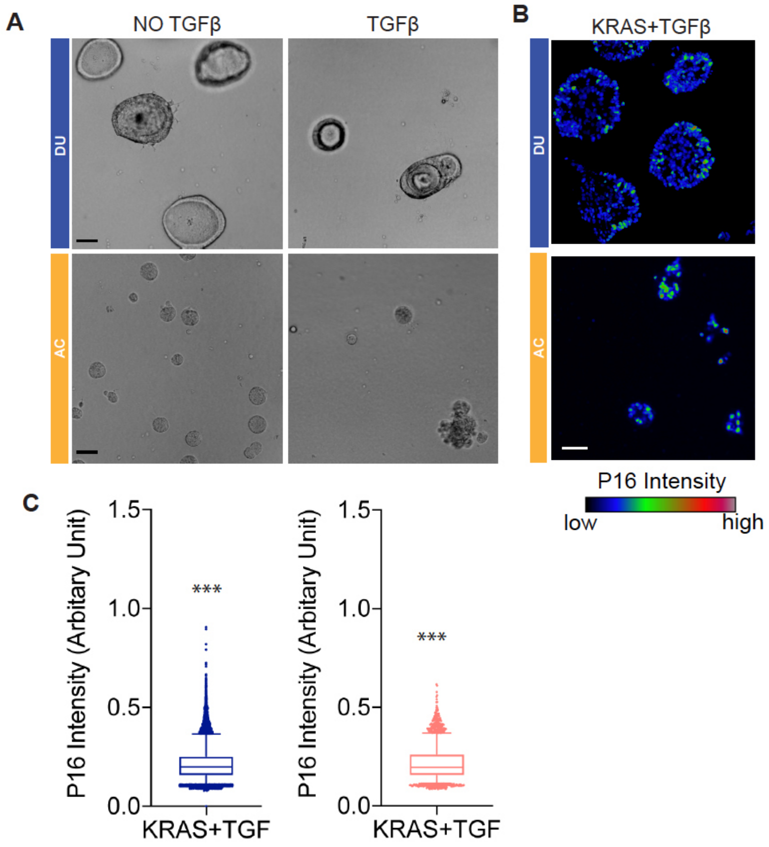

Figure S6. TGFβ treatments induce biological changes in organoids (related to Figure 6) **(A)** Phase contrast images of duct-like and acini-like organoids without and with TGFβ treatments. Scale bar, 50 µm. (**B)** Pseudocolor images indicating p16^{INK4A} intensities in *KRAS*^{G12D} expressing organoids with TGFβ treatments. Images converted from immunofluorescent staining of p16^{INK4A}. Scale bars, 100 µm. **(C)** Quantification of p16^{INK4A} expression per nuclei in *KRAS*^{G12D} organoids with TGFβ treatments (N>2500, from three independent cultures). Y-axis present signals normalized to maximal values of 8 bit image (255). Whisker-box plot, range 5-95%; center lines indicate median values; grey dots represent individual measurements. ***, p<0.001.

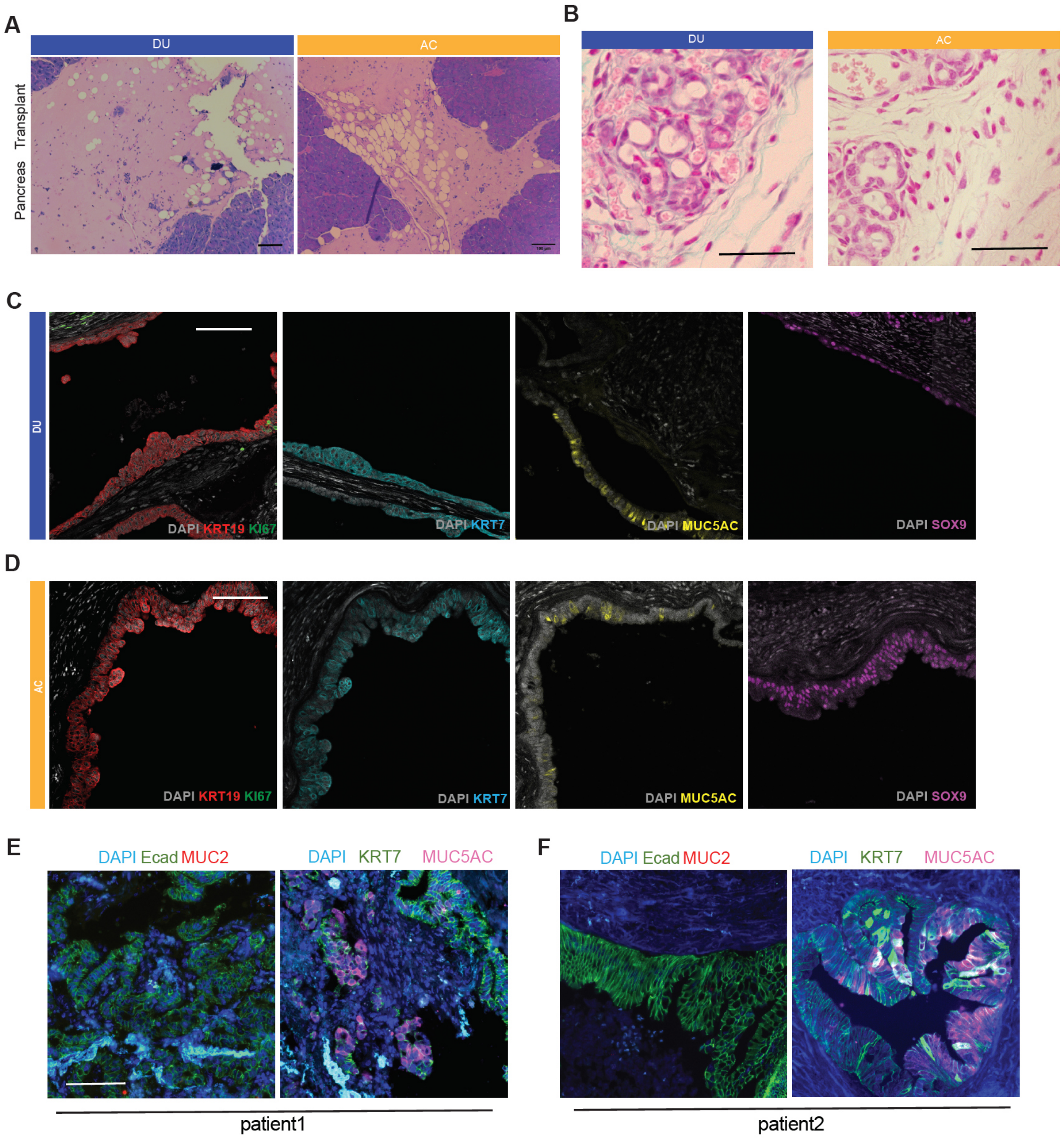

Figure S7. Orthotopic transplantation of organoids into mice (related to Figure 7) (A) H&E images of mouse pancreas transplanted with duct-like (DU) and acini-like organoids (AC) without oncogene expression (N=10 mice for each group). Scale bars, 100 µm. **(B)** Mouse pancreatic tissues adjacent to organoid-derived lesions exhibiting pancreatitis-like histology. Scale bars, 100 µm. **(C-D)** Protein expression in lesions grown from $KRAS^{G12D}$ expressing duct-like (C) (N=9 mice) and acini-like (D) (N=10 mice) organoids. KRT19, red; Ki67, green; KRT7, teal; MUC5AC, yellow; SOX9, purple. Scale bars, 100 µm. **(E-F)** Protein expression in human PDAC tumor tissues. Left panel: E-cadherin, green; MUC2, red; DPAI, blue. Right panel: KRT7, green; MUC5AC, red.
