# Supplementary material for: Commitment and oncogene-induced plasticity of human stem cell-derived pancreatic acinar and ductal organoids
Source: Cell Stem Cell. 2021 Jun 3;28(6):1090–1104.e6. doi: 10.1016/j.stem.2021.03.022 (PMC8202734; doi:10.1016/j.stem.2021.03.022)
Supplement: Document S2. Article plus supplemental information [file mmc5.pdf]

Cell Stem Cell

Article

Commitment and oncogene-induced plasticity of human stem cell-derived pancreatic acinar and ductal organoids

Graphical abstract

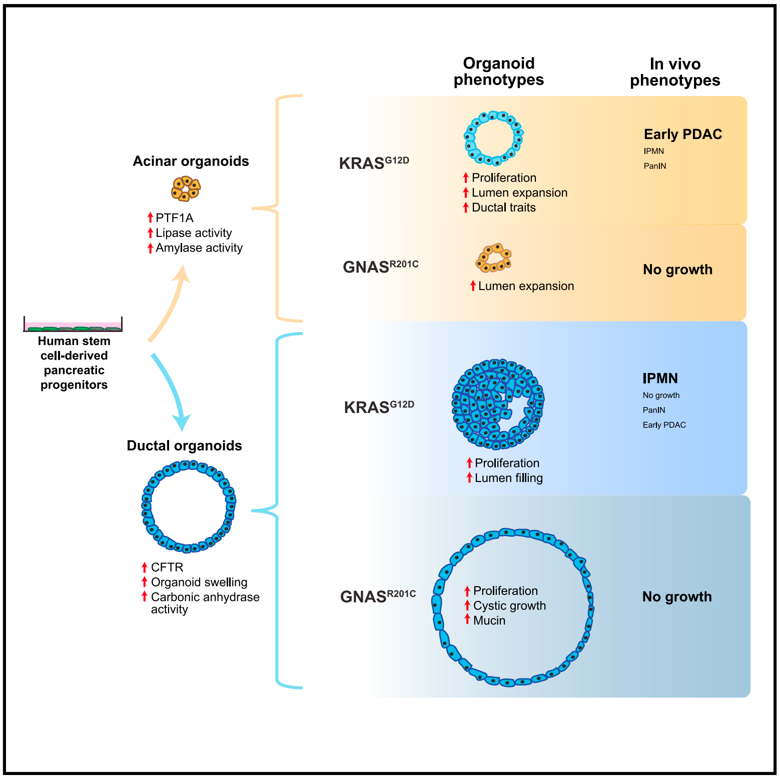

Authors

Ling Huang, Ridhdhi Desai, Daniel N. Conrad, ..., Lakshmi B. Muthuswamy, Zev Gartner, Senthil K. Muthuswamy

Correspondence

smuthusw@bidmc.harvard.edu

In brief

Huang et al. describe conditions for generation of pancreatic ductal and acinar organoids from human stem cells. Expression of KRAS^{G12D} or GNAS^{R201C} induced cell-lineage-specific phenotypes and cell-state plasticity in culture and *in vivo*, identifying a renewable source of ductal and acinar organoids for modeling development and diseases of the exocrine pancreas.

Highlights

- Generation and long-term maintenance of human acinar organoids from stem cells
- GNAS^{R201C} induces cystic growth more effectively in ductal than in acinar organoids
- KRAS^{G12D} induces cancerous lesions more often from acinar versus ductal organoids
- KRAS^{G12D} in acinar organoids induces an acinar-to-ductal metaplasia-like phenotype

Huang et al., 2021, Cell Stem Cell *28*, 1090–1104
June 3, 2021 © 2021 The Authors. Published by Elsevier Inc.
https://doi.org/10.1016/j.stem.2021.03.022

CellPress
OPEN ACCESS

Cell Stem Cell

Article

Commitment and oncogene-induced plasticity of human stem cell-derived pancreatic acinar and ductal organoids

Ling Huang,[1,2] Ridhdhi Desai,[1,2] Daniel N. Conrad,[3] Nayara C. Leite,[4] Dipikaa Akshinthala,[1,2] Christine Maria Lim,[1,2] Raul Gonzalez,[1,5] Lakshmi B. Muthuswamy,[1,2] Zev Gartner,[3,6,7] and Senthil K. Muthuswamy[1,2,8,*]

[1]Cancer Research Institute, Beth Israel Deaconess Medical Center, Harvard Medical School, Boston, MA 02215, USA
[2]Department of Medicine, Beth Israel Deaconess Medical Center, Harvard Medical School, Boston, MA 02215, USA
[3]Department of Pharmaceutical Chemistry, University of California, San Francisco, San Francisco, CA 94158, USA
[4]Department of Stem Cell and Regenerative Biology, Harvard University, Cambridge, MA 02138, USA
[5]Department of Pathology, Beth Israel Deaconess Medical Center, Harvard Medical School, Boston, MA 02215, USA
[6]Chan-Zuckerberg Biohub, San Francisco, CA 94158, USA
[7]NSF Center for Cellular Construction, San Francisco, CA 94158, USA
[8]Lead contact
*Correspondence: smuthusw@bidmc.harvard.edu
https://doi.org/10.1016/j.stem.2021.03.022

SUMMARY

The exocrine pancreas, consisting of ducts and acini, is the site of origin of pancreatitis and pancreatic ductal adenocarcinoma (PDAC). Our understanding of the genesis and progression of human pancreatic diseases, including PDAC, is limited because of challenges in maintaining human acinar and ductal cells in culture. Here we report induction of human pluripotent stem cells toward pancreatic ductal and acinar organoids that recapitulate properties of the neonatal exocrine pancreas. Expression of the PDAC-associated oncogene GNAS^{R201C} induces cystic growth more effectively in ductal than acinar organoids, whereas KRAS^{G12D} is more effective in modeling cancer *in vivo* when expressed in acinar compared with ductal organoids. KRAS^{G12D}, but not GNAS^{R201C}, induces acinar-to-ductal metaplasia-like changes in culture and *in vivo*. We develop a renewable source of ductal and acinar organoids for modeling exocrine development and diseases and demonstrate lineage tropism and plasticity for oncogene action in the human pancreas.

INTRODUCTION

The pancreas is composed of endocrine and exocrine compartments. Although the endocrine pancreas harbors islets of Langerhans, the exocrine pancreas, which makes up over 90% of the total pancreas volume, contains the ductal (~5%) and acinar (~85%) epithelia. The most common type of pancreatic cancer, pancreatic ductal adenocarcinoma (PDAC), is thought to arise from the exocrine compartment (Kleeff et al., 2016). Precursor lesions of pancreatic cancer can be classified into four main subtypes based on their clinical pathology: pancreatic intraepithelial neoplasia (PanIN), intraductal papillary mucinous neoplasms (IPMNs), intratubular papillary neoplasms (ITPNs), and mucinous neoplasms (MCNs) (Cooper et al., 2013; Hruban et al., 2000, 2007). Genomic studies reveal that *KRAS* mutations are observed in more than 80% of PanINs and associated with invasive PDAC (Almoguera et al., 1988; Kanda et al., 2012; Maitra and Hruban, 2008), identifying *KRAS* as the dominant driver gene in PDAC. Mutation in *GNAS* involving codon 201 is observed frequently in IPMN lesions, either by itself or in combination with mutant *KRAS* (Amato et al., 2014; Fukayama et al., 1986; Wu et al., 2011), and adjacent invasive PDAC, identifying *GNAS* as a driver in IPMN-derived PDAC. It is unclear how different precancerous lesions affect PDAC development and how the cellular origins of PDAC affect development of precancerous lesions and clinical prognosis.

Among the pancreatic cancer-associated mutations in *KRAS*, G12D is the most frequent (Waters and Der, 2018). In mouse models, multiple early studies using expression of mutant *KRas^{G12D}* alone or in combination with loss of the tumor suppressors *Tp53* or *Cdkn2a* under control of the *Pdx1* promoter resulted in development of PanIN-like lesions that progressed to PDAC (Aguirre et al., 2003; Bardeesy et al., 2006; Hingorani et al., 2003). *Pdx1* is expressed in progenitors of all pancreatic epithelia; hence, these studies do not provide direct insights into the cells of origin for PDAC. Expression of *KRas^{G12D}* under multiple acinar cell-specific promoters (*Mist1*, *Pftf1a*) supports an acinar cell of origin for PDAC (De La O et al., 2008; Gidekel Friedlander et al., 2009; Habbe et al., 2008; Tuveson et al., 2006), where acinar cells undergo ductal metaplasia early during the tumorigenic process. Compelling evidence generated by Sanders, Kopp, and Behrens provided additional insights

 © 2021 The Authors. Published by Elsevier Inc.
This is an open access article under the CC BY license (http://creativecommons.org/licenses/by/4.0/).

(Ferreira et al., 2017; Kopp et al., 2012; Lee et al., 2019). Expression of *KRas*^{G12D} under acinar cell-specific *Ptf1a*-Cre induced PanINs more readily than under duct-selective *Sox9*-Cre (Kopp et al., 2012), suggesting that acinar cells are more sensitive to *KRAS*-induced PanIN initiation. Combining *KRas*^{G12D} expression with loss of *Tp53* or *Fbw7* under control of acinus-specific elastase or *Ptf1a*-Cre induces PanIN lesions that progress to PDAC (Ferreira et al., 2017; Lee et al., 2019); however, the same gene combinations under control of ductal cell-specific *Sox9* or *Krt19*-Cre results in PanIN-independent development of PDAC that was more aggressive, suggesting a distinct evolutionary path for ductal epithelium-initiated cancers (Ferreira et al., 2017; Lee et al., 2019). The existence of different paths for acinus-initiated and duct-initiated PDAC tumorigenesis highlights the importance of cells of origin during initiation and progression of PDAC.

IPMN is associated anatomically with ducts in the human pancreas; however, studies with duct-specific expression of *GNAS* mutants have not yet been reported. Expression of a PDAC-associated mutant of *Gnas*, *Gnas*^{R201C}, and *Kras*^{G12D} under control of the *Ptf1a* promoter is sufficient to induce dilation of pancreatic ducts and loss of acini, representing the IPMN phenotype (Ideno et al., 2018; Patra et al., 2018; Taki et al., 2016). Although it is not known whether duct-specific expression of a *GNAS* mutant is sufficient to induce IPMN lesions, *Pten* loss or *Lkb1* inactivation with *KRas* mutant expression in ductal epithelia drives IPMN-like lesions (Collet et al., 2020; Kopp et al., 2018), suggesting that IPMN-like lesions can be derived from ductal and acinar lesions in mice.

Modeling cancer initiation and progression in a human cell context will provide new biological insights that can be exploited for identification of biomarkers and treatments for PDAC. Ductal exocrine cells from the adult pancreas have been maintained successfully in culture (Boj et al., 2015; Huch et al., 2013; Seino et al., 2018) and engineered to express PDAC-associated mutations in *KRAS*, *CDKN2A*, *SMAD4*, and *TP53* to induce cell proliferation *in vitro* and form PanIN-like lesions *in vivo* without progression to PDAC (Lee et al., 2017). Efforts to maintain human acinar cells in culture and use them to model cancer initiation has been challenging because acinar cell cultures are short lived or undergo *trans*-differentiation by a process referred to as acinar-to-ductal metaplasia (ADM) to assume a duct-like state (De Lisle and Logsdon, 1990; Rooman et al., 2000).

Human pluripotent stem cells (hPSCs) are a powerful platform to generate multiple cell types in culture (Sharma et al., 2020). We and others have reported the ability to induce hPSC-derived pancreatic progenitor (PP) cells toward the exocrine lineage in culture, supporting formation of ductal and acinar structures when transplanted into the mouse pancreas (Hohwieler et al., 2017; Huang et al., 2015; Simsek et al., 2016). Conditions to generate populations of pancreatic ductal or acinar cells *in vitro* are challenging. Although murine embryonic stem cells can be induced to differentiate into acinar cells (Rovira et al., 2008), whether such strategies can be used to generate human pancreatic acinar cells is not known. This study reports our ability to generate pancreatic duct-like or acinus-like organoid structures from human stem cell-derived PP cells. The two lineages of organoids exhibit distinct morphologies, express lineage-enriched markers, and have lineage-specific enzyme activities. Using these organoids, we demonstrate that *GNAS*^{R201C} and *KRAS*^{G12D} exert lineage-specific effects in culture and *in vivo*.

RESULTS

Commitment of human PP cells toward acinar and ductal lineages

PDX1-positive PP cells obtained with an established protocol (Pagliuca et al., 2014) were used to generate ductal and acinar lineage-committed organoids in Matrigel-based cultures. To design the culture media, we focused on pathways with established roles in exocrine pancreas specification. For example, in the mouse embryonic pancreas, β-catenin was required for specification of pancreatic acinar cells, whereas it was largely dispensable for ductal and endocrine cells (Keefe et al., 2012). Continuous activation of the Notch signaling pathway promotes duct differentiation and suppresses acinar cell fate (Esni et al., 2004; Murtaugh et al., 2003; Shih et al., 2012). In addition, histone deacetylase (HDAC) inhibition suppresses the acinar fate and promotes ductal specification (Haumaitre et al., 2008). We methodically screened multiple combinations of factors and small molecules and optimized a protocol to induce differentiation of PP cells toward ductal or acinar lineage-specified organoids (see Figure 1A for a schematic). Specification of duct-like organoids was promoted by activation of the fibroblast growth factor (FGF), epidermal growth factor (EGF), non-canonical WNT family, and retinoic acid pathways and inhibition of the HDAC, canonical WNT family, and ALK5 pathways. On the other hand, specification of acinus-like organoids was promoted by activation of the canonical WNT, FGF, and cortisol pathways and inhibition of the hedgehog, NOTCH family, bone morphogenetic protein (BMP), and ALK5 pathways. Under these culture conditions, acinar and ductal organoids formed with 6.57% ± 0.23% and 1.7% – 0.24% efficiency (n = 3), respectively. Cells that did not develop into organoids died within 2 days in culture.

The acinar organoids were small (~20–105 μm in diameter on day 16) with no visible lumen. In comparison, ductal organoids were large (~50–220 μm in diameter on day 16) and cystic with visible hollow lumens (Figure 1B). Morphologically, both types of organoids increased their sizes during 3D growth and became spherical, as indicated by increases in organoid areas and form factor (a form factor of 1.0 is a perfect circle) (Figure 1C). Lumen expansion of ductal organoids started from day 8 in culture, and the majority of these organoids had a visible lumen by day 12. Both types of organoids were polarized, as indicated by the apical localization of the tight junction protein ZO1 and basal localization of the basement membrane protein collagen IV (Figure 1D). We also investigated subcellular features of organoids by transmission electron microscopy (TEM) (Figure 1E). TEM analysis showed that acinus-like organoids were compact with a small lumen, whereas duct-like organoids were large with a dilated lumen (Figure 1E). Cells in acinar organoids had many secretory vesicles located at the apical side (red arrow, Figure 1E); these vesicles were 0.5–1.0 μm in diameter and slightly electron dense. Although these vesicles did not resemble the apically localized zymogen granules present in mature human acinar cells (Lugea et al., 2017), they were similar to precursors of zymogen granules observed in 12- to 14-week-old human fetal pancreata (Laitio

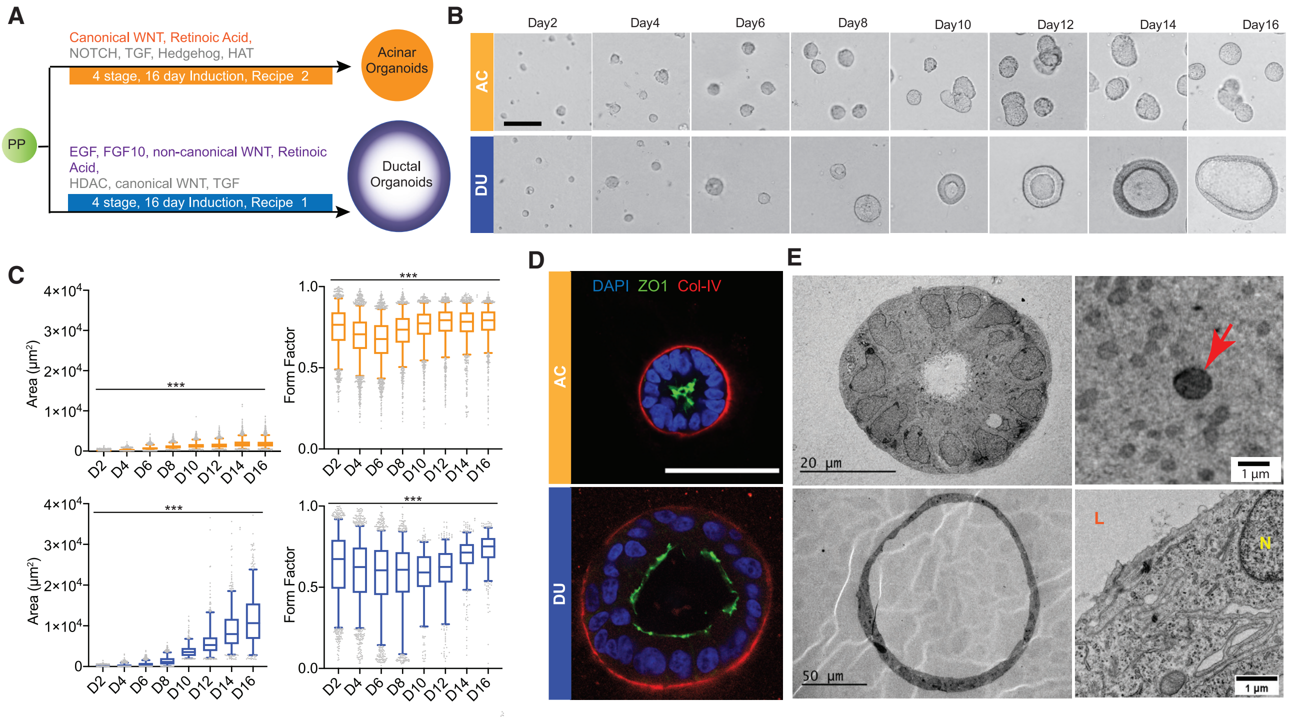

Figure 1. Commitment of human PP cells toward the acinar and ductal lineages

(A) Schematic of the duct-like and acinus-like organoid induction protocols.

(B) Phase-contrast images of organoids during 16-day culture (n = 3, independent cultures). Scale bar, 50 μm.

(C) Changes in total area and form factor (circularity index) of organoids during morphogenesis (n > 150, n = 3 independent cultures). Box-and-whisker plot, range 5%–95%; center lines indicate median values; gray dots represent individual measurements. ***$p < 0.001$.

(D) Immunostaining for collagen IV (red), DAPI (blue), and ZO1 (green). Scale bar, 50 μm.

(E) TEM images of acinar and ductal organoids. Red arrow, electron-dense vesicles; N, nucleus; L, lumen. DU (blue), duct-like organoid; AC (orange), acinus-like organoid.

et al., 1974), suggesting that our acinus-like organoids resembled fetal and not mature pancreatic acini. PP cells generated from two independent human induced pluripotent stem cells (iPSCs) (see Figure S1A for details) also formed acinar and ductal organoids with morphological features comparable with organoids obtained from human embryonic stem cell (hESC)-derived PP cells (Figures S1B and S1C), demonstrating that the condition we developed was effective in supporting differentiation of PP cells generated from hESC and iPSC lines.

Single-cell analysis of hESC-derived duct-like and acinus-like organoids

We used single-nucleus RNA (snRNA) sequencing as an unbiased assessment of the molecular differences between the two organoid lineages. Samples were multiplexed in the 10X Chromium system to obtain single-nucleus transcriptomes from PP cells and day 8 acinar and ductal organoids (McGinnis et al., 2019). Uniform manifold approximation and projection (UMAP) visualization of the data revealed three clusters of cells; each was enriched for cells from one of the three samples, and the replicates clustered together (Figure 2A). Of the common genes shared by two cell clusters, ductal organoids and progenitor cells exhibited the greatest overlap (Figure 2B), suggesting that acinar organoids were more divergent from progenitor cells. To assess the unique characteristics of each cell type, we re-computed markers to identify genes specifically enriched in each cluster (Table S1). Some classical exocrine lineage markers, including *SOX9*, *HNF1B*, *CEL*, *PNLIP*, *CTRB1*, and *CTRC*, showed trends in average expression across culture types that were in line with organoid differentiation (Figure S2A) but were not statistically different between cell groups. It is possible that use of nuclear RNA, because of technical challenges we experienced during isolation of cellular RNA from acinar organoids, contributed to the low signal.

The top 20 differentially upregulated or downregulated genes detected in our snRNA analysis (Table S2) were not widely studied markers for ductal and acinar cells of the pancreas. To evaluate their localization in the human pancreas, we checked expression patterns in the Human Protein Atlas (Uhlén et al., 2015; Table S3). CALN1, CRID2, DLG2, and LRP1B, enriched in acinar organoids, showed localization patterns consistent with acinar cells in the human pancreas (Figures S2B–S2E), and SLC4A4, ANXA4, C8orf34, and MAGI1, enriched in duct-like organoids, showed localization patterns consistent with ductal cells in the human pancreas (Figures S2F–S2I), further supporting the lineage commitment of hESC-derived acinus-like and duct-like organoids. The ratio of *RBPJL*/*RBPJ*, an indicator of acinar specification (Masui et al., 2010), was also highest in acinus-like organoids (Figure S2A). These results suggest that ductal organoids and acinar organoids adopt distinct differentiation paths.

CellPress
OPEN ACCESS

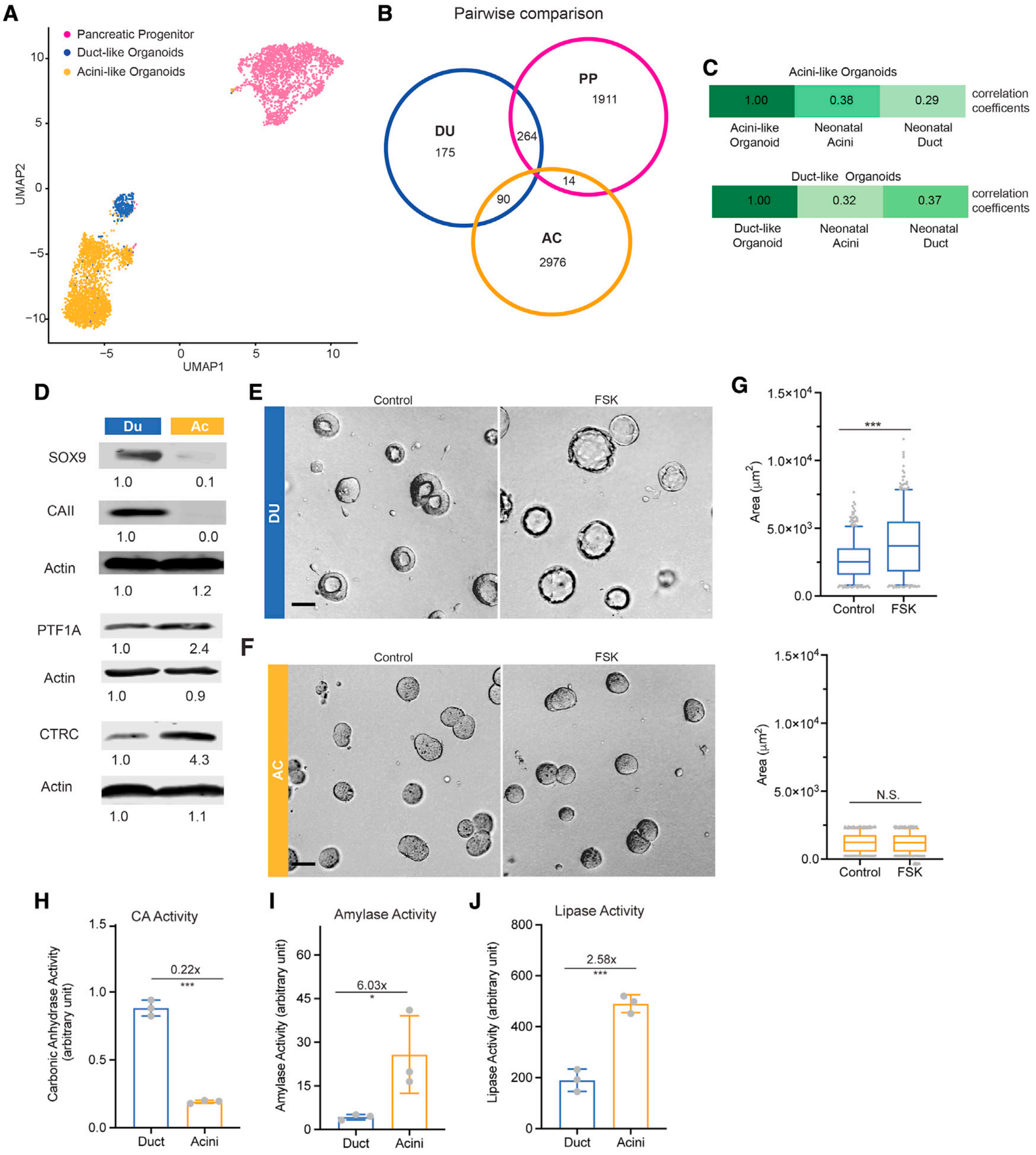

Figure 2. Single-cell analysis of hESC-derived duct-like and acinus-like organoids and expression of lineage markers and functional readouts

(A) Uniform manifold approximation and projection (UMAP) of single-nucleus transcriptomes of cells in pancreatic progenitors (PPs; pink), DUs (blue) and ACs (orange). Shown are results from two independent cultures and sequencing.

(B) Venn diagram of cell type markers identified by pairwise comparison using adjusted $p \leq 0.05$ and average log(fold-change) > 0. The color scheme is the same as in (A).

(C) Comparison of Pearson correlation coefficients for expression of marker genes (1,012 genes; adjusted (adj.) $p < 0.05$) between organoid culture and human neonatal acinar and ductal cells. Marker genes were derived by identifying differentially expressed genes ($p < 0.05$) in cells under different culture conditions. Wilcoxon rank-sum test was used.

(D) Immunoblot analysis for acinar and ductal markers (n = 3 independent cultures). The numbers under the blots represent normalized signals of protein bands.

(E and F) Morphological changes of day 8 DUs (blue) and ACs (orange) in response to 10 μM forskolin treatment (2-h incubation). Scale bar, 50 μm.

(G) Measurements of organoid size changes induced by forskolin treatment. Box-and-whisker plot, range 5%–95%; center lines indicate median values; gray dots represent individual measurements (n > 200, combination of three independent cultures).

(H–J) Measurement of enzyme activities in DU and AC lysates (n = 3 independent cultures). y axis, enzyme activity in arbitrary units.

Error bars in bar charts represent standard deviation; gray dots represent individual measurements. ***$p < 0.001$; *, $0.01 < p < 0.05$; N.S., not significant, $p > 0.05$.

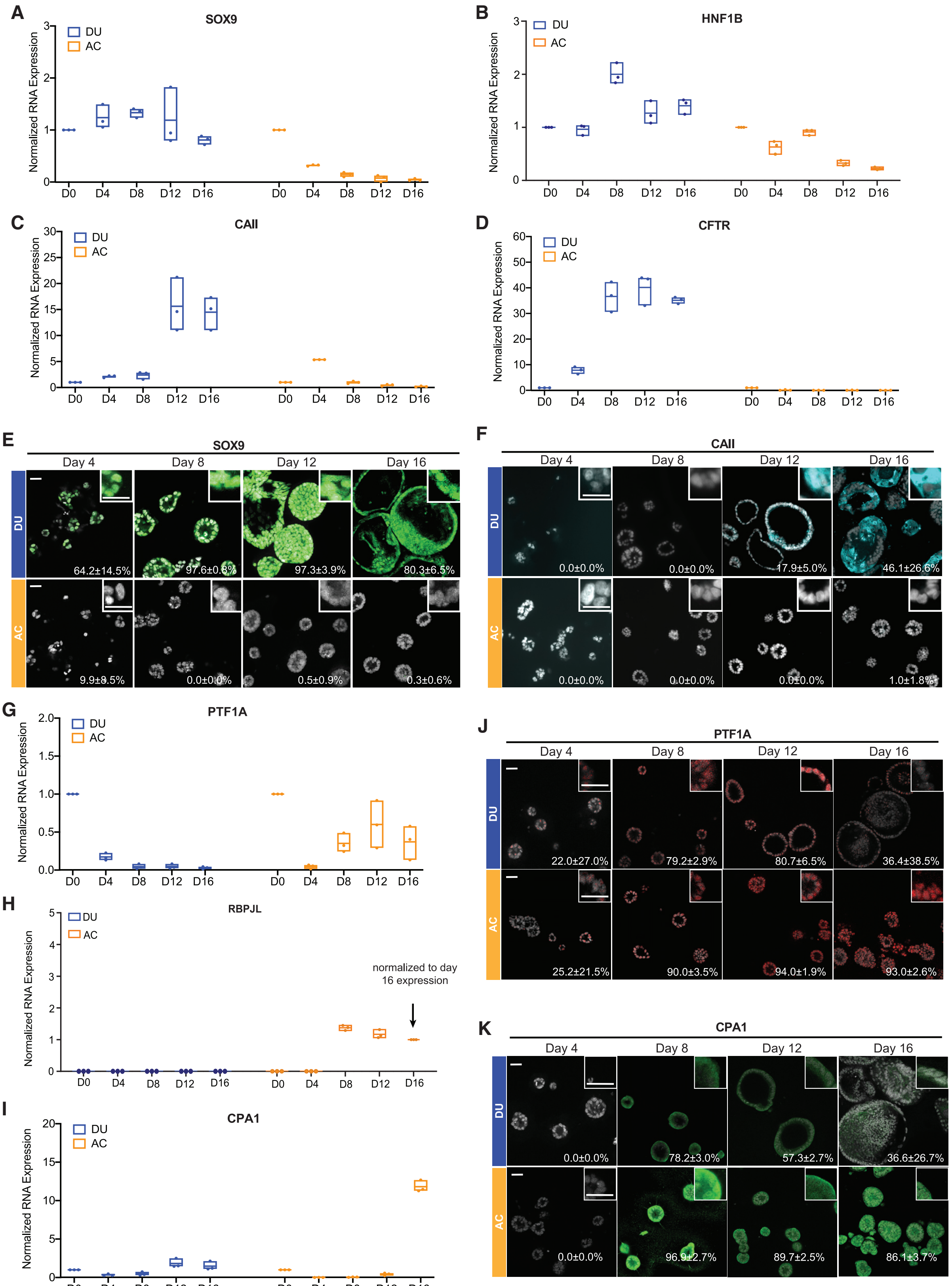

(legend on next page)

To contextualize the relative differentiation of our organoids compared with human acinar and ductal cells, we downloaded one published snRNA sequencing dataset of human pancreas tissue (Tosti et al., 2021). Using the average expression profiles of marker genes derived from our organoids and progenitors, we calculated Pearson correlation coefficients for our acinar and ductal organoids and the acinar and ductal cells from the neonatal dataset (Figure 2C). The trends in these correlations are consistent with the notion that, by day 8, duct-like and acinus-like organoid cultures were induced toward more duct- or acinus-like fates, respectively. Given the correlations with the neonatal exocrine pancreas and the low representation of genes expressed in adult ductal and acinar cells, our stem cell-derived organoids are likely to represent more neonatal states; hence, we refer to them as "acinus-like" and "duct-like" organoid structures.

Expression of acinar and ductal lineage markers and functional readouts

Next we took a candidate approach to monitor the expression of recognized ductal and acinar markers in our acinus-like and duct-like structures. Protein expression of the pancreatic ductal markers SOX9 and carbonic anhydrase II (CAII) and the pancreatic acinar markers PTF1A and chymotrypsin C (CTRC) (Figure 2D) demonstrated that the duct-like organoids expressed high levels of SOX9 and CAII, which were undetectable or very low in acinus-like organoids. In contrast, acinus-like organoids expressed PTF1A and CTRC at higher levels compared with duct-like organoids.

To assess functional differentiation, the organoids were treated with 10 μM forskolin for 2 h to monitor changes in swelling. Duct-like but not acinus-like organoids showed a significant expansion of the luminal space (Figures 2E–2G). Forskolin is known to induce luminal expansion in a cystic fibrosis transmembrane conductance regulator (CFTR)-dependent manner and loss of CFTR expression or cells with inactive mutants of CFTR fail to respond to forskolin treatment (Dekkers et al., 2013). The differential response to forskolin treatment by our duct-like and acinus-like organoids demonstrates functional CFTR in duct-like organoids and further supports ductal lineage commitment. In addition, carbonic anhydrase activity was four times higher in duct-like organoids compared with acinus-like organoids (Figure 2H). In contrast, amylase and lipase activities (associated with acinar cells) were more elevated in acinus-like organoids compared with duct-like organoids, demonstrating lineage-specific functions (Figures 2I and 2J). Acinus-like organoids derived from iPSCs also had lower expression of *PDX1*, *CFTR*, and *NKX6.1* compared with duct-like organoids (Figure S3A). PTF1A expression in acinus-like organoids derived from iPSC line 11 was also higher than in duct-like organoids (Figure S3A).

Temporal changes in marker expression during duct-like and acinus-like lineage specification

To better understand the timeline of lineage commitment, we monitored the expression of ductal (*SOX9*, *HNF1B*, and *CAII*) and acinar (*PTF1A*, *RBPJL*, and *CPA1)* markers over time (Figure 3). *HNF1B* and *SOX9* expression was high in PP cells and remained high during morphogenesis in duct-like organoids (Figures 3A, 3B, and 3E), whereas they decreased over time in acinus-like organoids (Figures 3A, 3B, and 3E). *CAII* RNA and protein and *CFTR* RNA expression increased from day 8 and was upregulated significantly by day12 during ductal differentiation but not during acinar differentiation (Figures 3C, 3D, and 3F).

Expression of *PTF1A* RNA and proteins in acinus-like organoids increased beginning on day 8 and remained high, whereas the levels decreased progressively during ductal differentiation (Figures 3G and 3J). *RBPJL* expression is associated with the acinar cell type (Masui et al., 2010). *RBPJL* RNA levels were high in acinar cultures starting on day 8 but undetectable in duct-like organoids (Figure 3H). However, *CPA1*, the acinar cell enzyme, was expressed in acinus-like and duct-like organoids from day 8 (Figures 3I and 3K). RNA expression of the PP marker *PDX1* remained constant in duct-like organoids and was suppressed in acinus-like organoids (Figure S3B). Detectable expression of *CPA1* in our duct-like organoids is consistent with findings that embryonic ducts are plastic and can give rise to acinar cells (Reichert and Rustgi, 2011). The above results demonstrate that our culture conditions support commitment of PPs toward the acinus-like or duct-like lineage.

The *GNAS* mutant shows tropism for ductal-lineage epithelia

GNAS mutations occur frequently in cystic lesions of the pancreas (Ohtsuka et al., 2019). It is not known whether *GNAS* shows lineage tropism in human pancreatic cells. We engineered PP cells with doxycycline-inducible wild-type *GNAS* ($GNAS^{WT}$) or $GNAS^{R201C}$ (a mutation observed frequently in IPMN) and induced gene expression on day 8 of the differentiation protocol (Figure 4A). Expression of $GNAS^{R201C}$ promoted an increase in general phosphorylation of PKA substrates and phosphorylation of the specific PKA substrate vasodilator stimulated phosphoprotein (VASP) (Figure 4A). $GNAS^{R201C}$ was significantly better than $GNAS^{WT}$ in its ability to induce lumen expansion in duct-like organoids compared with acinus-like organoids (Figure 4B). Duct-like organoids expanded by about 8-fold in response to

Figure 3. Temporal changes in marker expression during duct-like and acinus-like lineage specification

(A–D) RNA expression of pancreatic ductal markers *SOX9* (A), *HNF1B* (B), *CAII* (C), and *CFTR* (D) in DUs and ACs during 16-day culture. Floating column charts represent RNA measurements from quantitative PCR, hinges represent maximal and minimal values, central lines indicate mean values, and dots represent individual measurements.

(E and F) Expression of SOX9 (E) and CAII (F) in organoids, detected by immunofluorescence. Numbers embedded in the images indicate the average percentage and standard deviation of marker-positive organoids. Scale bars, 50 μm.

(G–I) RNA expression of the pancreatic acinar markers *PTF1A* (G), *RBPJL* (H), and *CPA1* (I) in DUs and ACs during 16-day culture. Floating column charts: representation as in (A).

(J and K) Expression of PTF1A (J) and CPA1 (K) in organoids, detected by immunofluorescence. Numbers embedded in images indicate the average percentage and standard deviation of marker-positive organoids. Scale bars, 50 μm.

All results represent the sum of three independent cultures.

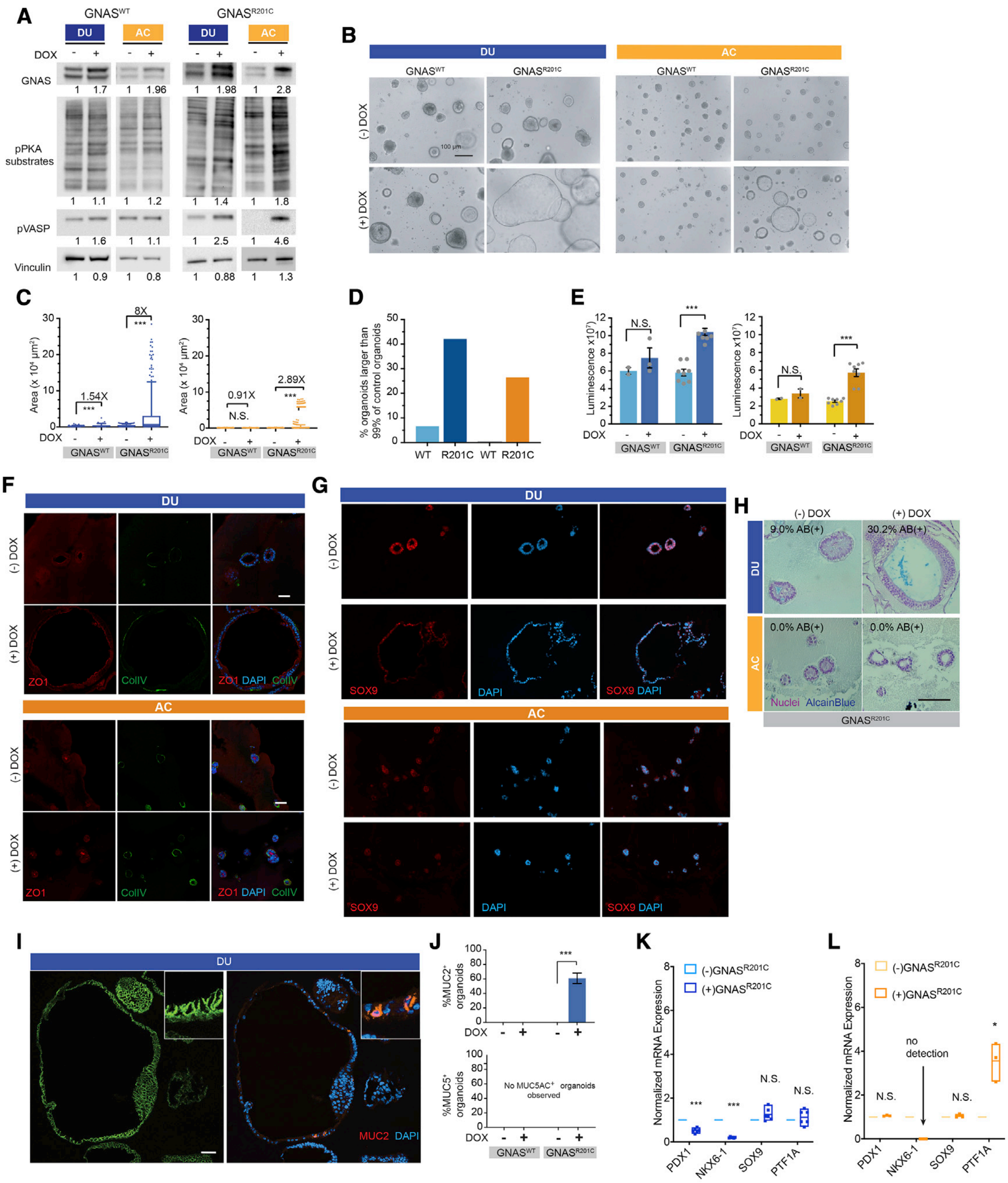

Figure 4. The GNAS mutant shows tropism for ductal-lineage epithelia

(A) Immunoblot analysis for GNAS expression and phosphorylation of GNAS downstream effectors in DUs and ACs (n = 3, independent cultures).

(B) Phase-contrast images of DUs and ACs expressing wild-type (WT) GNAS and $GNAS^{R201C}$. (-)DOX, without doxycline treatment; (+)DOX, with doxycycline treatment. Scale bar, 100 μm.

(C) Change in size, measured as the total area of organoids expressing $GNAS^{WT}$ or $GNAS^{R201C}$. Box-and-whisker plot, range 5%–95%; central lines, median values (n > 100, combination of three independent cultures).

(D) Percentage of organoids surpassing the 99th percentile of control organoid (no transgene expression) size (n = 3 independent cultures).

(E) Organoid cell number changes induced by $GNAS^{WT}$ and $GNAS^{R201C}$ as detected by CellTiter. Bar charts represent the mean and standard deviation of measurements from three independent cultures; gray dots represent individual measurements.

(F) Immunofluorescent staining of polarity proteins in organoids without (−DOX) and with (+DOX) $GNAS^{R201C}$ expression. ZO1, red; collagen IV, green; DAPI, blue. Scale bar, 100 μm.

(G) Expression of SOX9 in organoids without (−DOX) and with (+DOX) $GNAS^{R201C}$ expression. SOX9, red; DAPI, blue.

(H) AB (pH 2.5) staining of organoids without (−DOX) and with (+DOX) $GNAS^{R201C}$ expression. Scale bar, 100 μm.

(I) Expression of E-cadherin (green) and MUC2 (red) in DUs without (−DOX) and with (+DOX) $GNAS^{R201C}$ expression. Scale bar, 50 μm.

(legend continued on next page)

$GNAS^{R201C}$ expression, whereas acinar organoids showed only a 2.9-fold increase (Figure 4C). Overexpression of $GNAS^{WT}$ induced an ~1.5-fold expansion in duct-like organoids and no change in acinus-like organoids (Figure 4C). More than 40% of duct-like organoids expressing $GNAS^{R201C}$ were larger than 99% of the control organoids, whereas only 25% of acinus-like organoids expressing $GNAS^{R201C}$ were larger than 99% of the control organoids (Figure 4D), demonstrating that duct-like organoids were more sensitive than acinus-like organoids to $GNAS^{R201C}$ expression.

$GNAS^{R021C}$ but not $GNAS^{WT}$ expression increased cell numbers in organoid cultures (Figure 4E), with no detectable effect on localization of the cell polarity proteins ZO1 (an apical protein) and collagen IV (a basement membrane protein) (Figure 4F) or SOX9 in duct-like and acinus-like organoids (Figure 4G). To investigate whether *GNAS* induces changes in mucin overexpression, a hallmark of premalignant pancreas lesions, we stained structures with Alcian blue (an acidic mucin dye). More than 30% of duct-like organoids expressing $GNAS^{R201C}$ stained positive for Alcian blue (Figure 4H), with no detectable staining of acinus-like organoids. To better understand the patterns of mucin expression, we monitored changes in MUC2 and MUC5AC because these mucins are expressed in less than 4% of normal pancreatic cells (Kim et al., 2002) but are detected routinely in precursor lesions. Interestingly, MUC2 expression was observed in clusters of cells in about 60% of duct-like organoids expressing $GNAS^{R201C}$ (Figures 4I and 4J) but not in organoids expressing $GNAS^{WT}$ (Figure S4A). No MUC2 expression was detected in acinus-like organoids under any condition (Figures S4B and S4C). Neither duct-like nor acinus-like organoids expressed MUC5AC (Figures 4J, S4B, and S4C). These observations support our conclusion that human ductal epithelium is more sensitive to $GNAS^{R201C}$-induced changes than acinus-like cells.

Next we analyzed changes in expression of markers of PP cells (*NKX6.1*, *PDX1*), ductal (*SOX9*, *CFTR*), and acinar (*PTF1A*) epithelia (Figures 4K and 4L). Interestingly, the expression levels of *PDX1* and *NKX6.1* decreased in response to $GNAS^{R201C}$ expression in duct-like epithelia (Figure 4K). In acinar organoids, *NKX6.1* levels decreased to undetectable levels, and *PTF1A* levels increased (Figure 4L). *CFTR* was upregulated weakly by $GNAS^{R201C}$ in both lineages (Figure S4D).

Next we transplanted $GNAS^{R201C}$-expressing duct-like organoids into the mouse pancreas to investigate whether $GNAS^{R201C}$ expression was sufficient to induce precancerous lesions *in vivo*. Organoids were disassociated and injected into the pancreas of 6- to 8-week-old non-obese diabetic (NOD) CRISPR *Prkdc Il2r Gamma* (NCG) mice (N = 12). We did not observe any outgrowth that stained positive for human KRT19 or class I major histocompatibility complex (HLA) or human nuclear antigen. However, two of eight mice sacrificed after 8 weeks and one of four mice sacrificed after 14 weeks showed abnormal regions representing proliferative glands and metaplasia, likely because of injection-induced reactions (Figures S4E and S4F). The presence of the injection-site reaction in the pancreas validates the injection technique and supports the conclusion that $GNAS^{R201C}$ expression is not sufficient to support survival and growth of duct-like cells *in vivo*.

$KRAS^{G12D}$ induces ADM-like changes in acinus-like organoids but a progenitor phenotype in duct-like organoids

Mutations of *KRAS* are the most common genomic changes in precancerous lesions of PDAC (Buscail et al., 2020). *KRAS* is also overexpressed (Figure S5A**)** or amplified in human PDAC tumors (Mueller et al., 2018). To understand how oncogenic *KRAS* affects our human pancreatic acinus-like and duct-like lineages, we expressed $KRAS^{G12D}$ in PP cells in a doxycycline-inducible manner. We titrated the virus to allow ectopic expression of $KRAS^{G12D}$ within 2- to 4-fold of endogenous levels (Figures S5B and S5C) because a higher level of *KRAS* overexpression was not tolerated by these organoids (Figure S5D). In acinus-like and duct-like organoids, $KRAS^{G12D}$ expression induced a modest increase in phosphorylation of mitogen-activated protein kinase (p-ERK) (Figures S5B and S5C), consistent with previous studies (Gillies et al., 2020; Tuveson et al., 2004). Expression of $KRAS^{G12D}$ was induced on day 8 after cells were committed to a duct-like or acinus-like lineage, and the phenotypes were analyzed 16 days later. Consistent with previous reports (Serrano et al., 1997), expression of $KRAS^{G12D}$ induced a modest increase in expression of nuclear $p16^{INK4A}$ (Figures S5E and S5F), a senescence-associated marker. However, in duct-like and acinus-like organoids, $KRAS^{G12D}$ increased cell proliferation, as monitored by Ki67 positivity (Figures 5A and 5B) and total cell numbers (Figure 5C), suggesting that the level of expression of $KRAS^{G12D}$ is pro-proliferative and not high enough to induce senescent arrest. Morphologically, $KRAS^{G12D}$ induced a 4-fold increase in the number of structures with lumens filled by multilayering of cells, as monitored in phase-contrast and DAPI-stained images (Figures 5D and 5E). In contrast, expression of $KRAS^{G12D}$ induced a 5-fold increase in organoids with a visible lumen (Figures 5D and 5E) in acinar lineage-committed organoids. $KRAS^{G12D}$ induced no obvious increase in the overall size of duct-like organoids but a significant change in acinus-like organoids (Figure 5F). These observations demonstrate that, although $KRAS^{G12D}$ is equally competent in inducing cell proliferation in both lineages, it differentially affects the morphology of acinus-like and duct-like organoids.

To better understand the morphological changes in acinus-like organoids in response to expression of $KRAS^{G12D}$, we assessed changes in expression of markers of PP (*NKX6.1*, *PDX1*), ductal (*SOX9*, *CFTR*), and acinar (*PTF1A*, *CPA1*) epithelial cells. In duct-like organoids, $KRAS^{G12D}$ upregulated *PDX1*,

(J) Percentages of DUs expressing MUC2 (top chart) or MUC5AC (bottom chart). Bar charts represent the mean and standard deviation of measurements from three independent cultures; gray dots represent individual measurements.

(K and L) RNA expression of lineage markers in DUs (K) and ACs (L) altered by $GNAS^{R01C}$ expression. Floating column charts represent RNA measurements from quantitative PCR (n = 3 technical repeats), hinges represent maximal and minimal values, central lines indicate mean values, and dots represent individual measurements. ***p < 0.001; **, 0.001 < p < 0.01; *, 0. 01 < p < 0.05; N.S., p > 0.05.

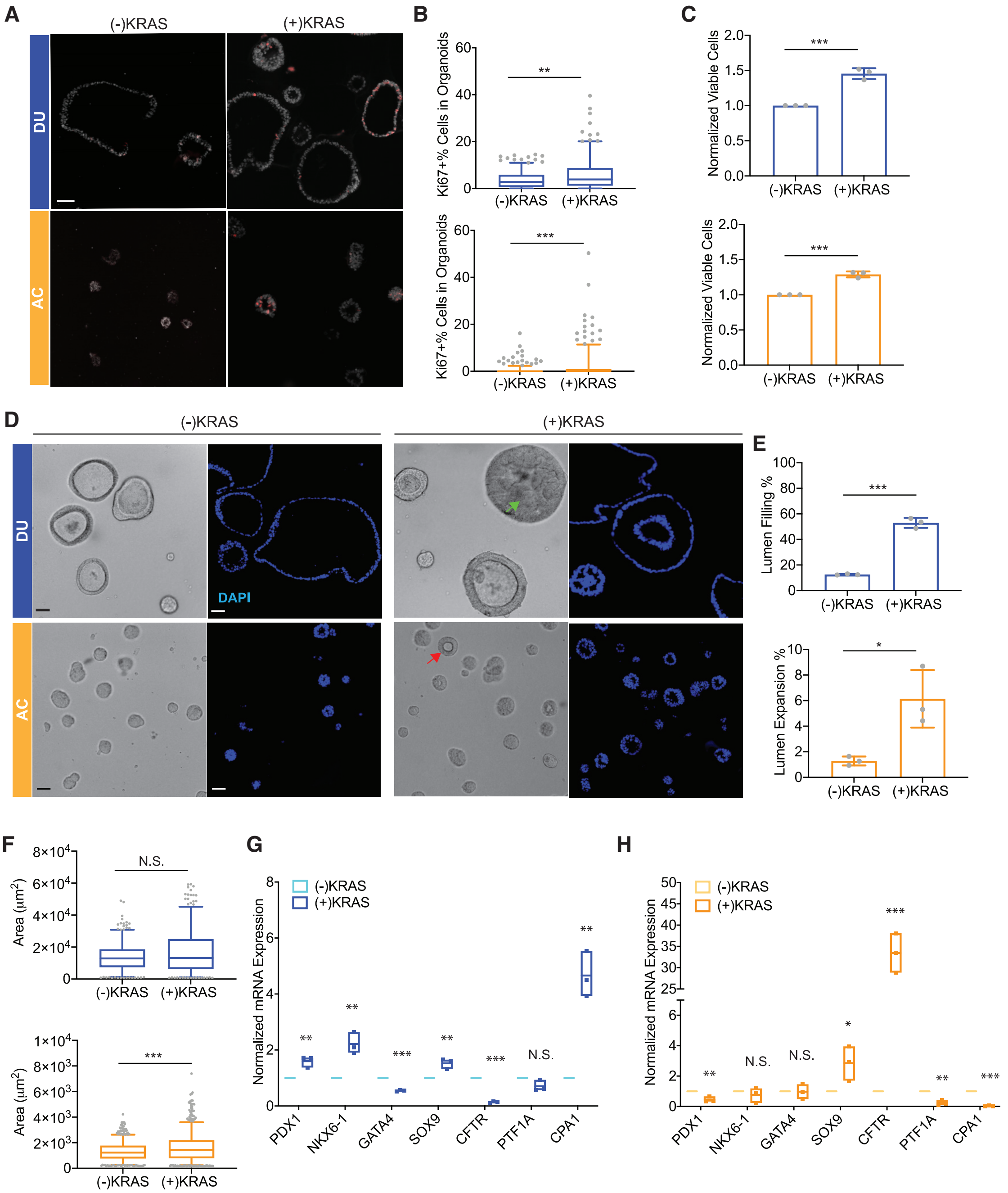

Figure 5. KRAS^{G12D} induces ADM-like changes in ACs but a progenitor phenotype in DUs

(A) Expression of the cell proliferation marker Ki67 (red) in DUs (blue) and ACs (orange) with and without KRAS^{G12D} expression. Scale bar, 50 μm.

(B) Quantification of Ki67-positive organoids with and without KRAS^{G12D} expression. Box-and-whisker plot, range 5%–95%; center lines indicate median values; gray dots represent individual measurements.

(C) Organoid cell number changes induced by KRAS^{G12D} expression. Bar charts represent the mean and standard deviation of measurements from three independent cultures; gray dots represent individual measurements.

(D and E) Quantification of organoid morphological changes induced by KRAS^{G12D} expression. Bar chart representation as in (C).

(F) Organoid size changes induced by KRAS^{G12D} expression. Box-and-whisker plot representation as in (B).

(G and H) Expression of pancreatic lineage markers in DUs (G) and ACs (H) altered by KRAS^{G12D} expression. Floating column charts represent RNA measurements from quantitative PCR, hinges represent maximal and minimal values, central lines indicate mean values, and dots represent individual measurements.
***$p < 0.001$; **, $0.001 < p < 0.01$; *, $0.01 < p < 0.05$; N.S., $p > 0.05$.
All results represent the sum of three independent cultures.

NKX6.1, and *SOX9* and downregulated and *CFTR*, suggesting change toward a progenitor state (Figure 5G). Interestingly, in acinus-like organoids, *KRAS*^{G12D} downregulated genes associated with acinar epithelia, *PTF1A* and *CPA1*, and upregulated *SOX9* expression (Figure 5H), suggesting *trans*-differentiation of an acinus-like into a duct-like state, recapitulating phenotypes of ADM.

Transforming growth factor β (TGF-β) augments *KRAS*^{G12D}-induced phenotypes in acinus-like and duct-like organoids

KRAS^{G12D} cooperates with cerulein-induced acute pancreatitis to initiate tumorigenesis in mouse models of PDAC (Guerra et al., 2007). Because TGF-β levels increase during acute pancreatitis (Riesle et al., 1997), and TGF-β induces *trans*-differentiation of exocrine pancreatic epithelial cells in culture (Handler et al., 2018; Liu et al., 2016), we investigated whether TGF-β cooperates with *KRAS*^{G12D} in duct-like and acinus-like organoids. TGF-β treatment did not alter expression of *KRAS*^{G12D} in organoids (Figure S5B). In the absence of *KRAS*^{G12D} expression, TGF-β induced cell death (Figure S6A) in acinus-like and duct-like organoids, whereas in *KRAS*^{G12D} expressing cells, TGF-β induced cell proliferation in ductal and acinar organoids by 1.7-fold and 2.4-fold, respectively (compare Figure 6A with Figures 5A and 5B), as well as suppression of *KRAS*^{G12D}-induced p16^{INK4A} expression (compare Figures S6B and S7C with Figures S5E and S5F). Morphologically, TGF-β treatment of *KRAS*^{G12D} expressing duct-like organoids induced a significant increase in the percentage of organoids with filled lumens (compare Figures 6B and 6C with Figure 5E). TGF-β induced a shrinkage in the size of duct-like organoids (compare Figure 6D with Figure 5F), likely because of compaction of cells and collapsing lumens (Figures 6B and 5D). In acinus-like organoids, TGF-β-induced an increase in organoid area and in the number of lumen-containing structures (compare Figures 6B–6D with Figures 5D and 5F).

In duct-like organoids, TGF-β further upregulated *NKX6.1* expression and suppressed *GATA4* and *PTF1A* expression compared with levels observed in the absence of TGF-β (Figures 6E and 5G). In acinus-like organoids, *KRAS*^{G12D} and TGF-β upregulated *PDX1*, *NKX6.1*, *PTF1A*, and *CPA1* (Figures 6F and 5H), suggesting reversion to a progenitor state. Prolonged stimulation of transformed epithelial cells with TGF-β is known to induce epithelial-to-mesenchymal transition (EMT) (Xu et al., 2009). Phase-contrast and H&E morphology analysis of *KRAS*^{G12D}-expressing organoids showed that TGF-β did not induce invasive behavior typically associated with EMT (Figure 6H). Although we cannot rule out acquisition of partial EMT states (Aiello et al., 2018), TGF-β-treated organoids showed an increase in the ductal epithelial marker KRT7 (Figure 6G), suggesting that the cells maintain their epithelial state under TGF-β stimulation conditions.

We studied the effect of *KRAS*^{G12D} and TGF-β on presentation of acidic mucins using Alcian blue (AB) staining. AB signals were increased by *KRAS*^{G12D} and enhanced further by TGF-β (Figure 6H). This effect was more dramatic in duct-like organoids than in acinus-like organoids. Among the mucins, expression of MUC5AC is associated with PanINs and all stages of pancreatic cancer (Kim et al., 2002), and a low frequency of MUC2 expression is associated with cystic lesions. Control organoids did not express either marker (Figures 6I and 6J), whereas MUC2 was detected in *KRAS*^{G12D}-expressing duct-like organoids with low frequency (~2.5%), and this was lost in TGF-β-treated organoids. MUC2 expression was not detected in acinus-like organoids (Figure 6I), regardless of treatment conditions. MUC5AC was rarely expressed in duct-like organoids with *KRAS*^{G12D} expression (Figure 6J) but expressed strongly in ~30% of *KRAS*^{G12D}-expressing organoids, suggesting a PanIN-like change.

KRAS^{G12D} is more effective in inducing acinus-like organoids to form early pancreatic-cancer-like lesions *in vivo*

To investigate *in vivo* phenotypes, duct-like or acinus-like organoids that were grown in the presence or absence of *KRAS*^{G12D} induction were dissociated and injected into the pancreata of 6- to 8-week old NCG mice at a rate of 500,000 cells per mouse. Immediately after surgery, mice were fed regular food (for organoids cultures without doxycycline (-DOX) ; without *KRAS*^{G12D}expression) or DOX-containing food (for organoids from the +DOX cultures; with *KRAS*^{G12D}expression) and monitored by palpation twice a week. All mice were sacrificed 8 weeks after transplantation.

We did not observe any transplant growth from control organoids (-DOX, N = 10; Figure S7A). In the presence of DOX, 67% of mice (7 of 10) transplanted with duct-like organoids expressing *KRAS*^{G12D} exhibited a wide spectrum of growth patterns, and three had no growth. Four had lesions with large, convoluted, dilated ductal structures lined with epithelium showing mild to moderate dysplastic changes, analogous to IPMN in humans (Figure 7A), and one mouse had a slightly dilated pancreatic duct, analogous to PanIN, and lesions with a convoluted conglomeration of duct structures in myoxid stroma lined with mucinous epithelium showing moderate to severe dysplastic change, analogous to early adenocarcinoma (Figure 7A). In contrast, 100% of mice (10 of 10) transplanted with acinus-like organoids expressing *KRAS*^{G12D} showed diverse patterns of growth *in vivo*. Seven mice had lesions exhibiting mild to moderate dysplastic changes, containing occasional goblet cells and focal cribriform, which were analogous to early adenocarcinoma in humans (Figure 7B). Two had lesions with dilated duct structures, analogous to IPMN (Figure 7B), and one mouse had a slightly dilated duct lined by normal serous epithelium, analogous to human PanIN (Figure 7B). Cells in lesions formed by duct-like and acinus-like organoids were positive for human cytokeratin 19, confirming that they were epithelial cells derived from human cells (Figures 7C, 7D, S7C, and S7D). Lesions with more aggressive histological phenotypes (Figures 7A and 7B) also had more frequent expression of Ki67 compared with those resembling precursor lesions (Figures 7C and 7D). In regions adjacent to normal mouse pancreas tissue, we observed histological phenotypes analogous to pancreatitis (Figures 7A and 7B, yellow arrows, and S7B). These adjacent regions, however, were negative for human cell markers and likely originated from mouse pancreatic cells.

Because *KRAS*^{G12D} expression altered cell differentiation of organoids *in vitro*, we also investigated cell differentiation in lesions

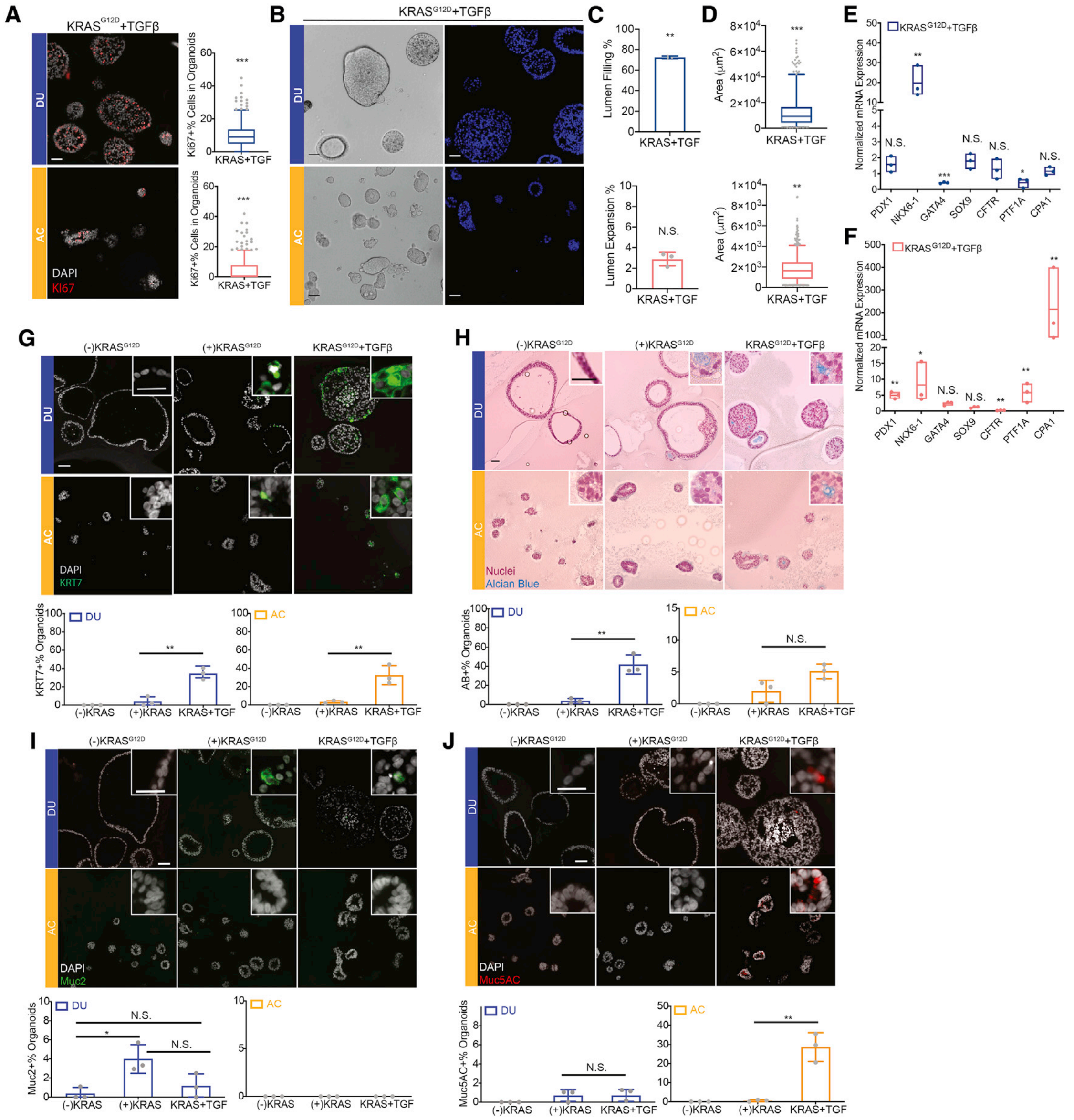

Figure 6. TGF-β augments KRAS^{G12D}-induced phenotypes in ACs and DUs

(A) Expression of the cell proliferation marker Ki67 (red) in DUs (blue) and ACs (orange) with KRAS^{G12D} expression and TGF-β treatment. Quantification of Ki67-positive organoids represented by box-and-whisker plots: range, 5%–95%; center lines indicate median values; gray dots represent individual measurement.

(B and C) Organoid morphological changes induced by KRAS^{G12D} expression and TGF-β treatment. Bar charts represent the mean and standard deviation of measurements from three independent cultures; gray dots represent individual measurements.

(D) Organoid size changes induced by KRAS^{G12D} expression and TGF-β treatment. Box-and-whisker plot representation as in (A).

(E and F) Expression of pancreatic lineage markers in DUs (E) and ACs (F) altered by KRAS^{G12D} expression and TGF-β treatment. Floating column charts represent RNA measurements from quantitative PCR (n = 3), hinges represent maximal and minimal values, central lines indicate mean values, and dots represent individual measurements.

(G–J) Detection of KRT7 (G), AB (H), MUC2 (I), and MUC5AC (J) in organoids under different experimental conditions. Bar chart representation as in (C).

Scale bars in main images, 50 µm; scale bars in insets, 25 µm. ***$p < 0.001$; **, $0.001 < p < 0.01$; *, $0.01 < p < 0.05$; N.S., $p > 0.05$. All results represent the sum of three independent cultures.

grown *in vivo*. Lesions grown from duct-like organoids had strong KRT7 and SOX9 expression (Figures 7C and S7C). Interestingly, lesions developed from acinus-like organoids also had strong KRT7 and SOX9 expression (Figures 7D and S7D), suggesting that the acinus-like cells with *KRAS^{G12D}* expression underwent *trans*-differentiation *in vivo* and shifted to a ductal fate. The lesions strongly expressed MUC5AC in a mosaic pattern (Figures 7C, 7D, S7C, and S7D), similar to what we observed in human PDAC samples (Figures S7E and S7F). These results demonstrate that both types of organoids could grow *in vivo*, recapitulating different stages of pancreatic tumorigenesis, with acinus-like organoids generating more aggressive lesions.

CellPress
OPEN ACCESS

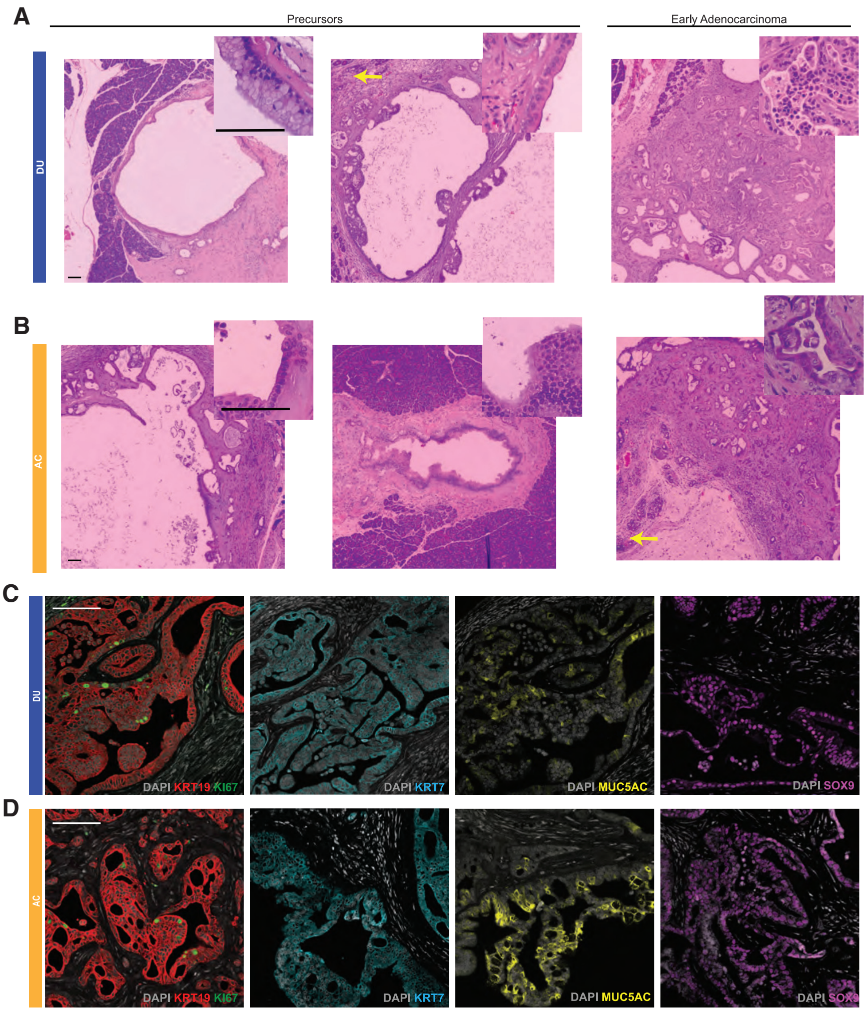

Figure 7. ACs expressing KRAS^{G12D} were more effective than DUs in inducing formation of early pancreatic-cancer-like lesions *in vivo*

(A and B) Histology of lesions grown from KRAS^{G12D}-expressing DUs (A, N = 9) or ACs (B, N = 10) transplanted into the mouse pancreas. Tissues were stained with nuclear red. Scale bars, 100 μm.

(C and D) Expression of human KRT19 (red), Ki67 (green), KRT7 (teal), MUC5AC (yellow), and SOX9 (purple) in lesions grown from KRAS^{G12D}-expressing DUs (C) or ACs (D). Scale bars, 100 μm.

DISCUSSION

We report the feasibility of generating pancreatic duct-like and acinus-like organoids resembling fetal or neonatal states from PP cells derived from hPSCs. Using these lineage-committed organoids, we demonstrate that *KRAS^{G12D}* and *GNAS^{R201C}* have lineage-specific effects in culture and *in vivo*. Our results are consistent with Breunig et al (Breunig et al., 2021) and together identify the pancreatic progenitor cell derived exocrine organoid platform as an opportunity to understand mechanisms regulating lineage commitment in the human exocrine pancreas and to model pancreatic cancer initiation and progression in culture.

Although acinus-like and ductal-like cells did not express all markers associated with adult pancreatic exocrine cells, the expression pattern we observed is consistent with human fetal exocrine pancreatic acinar cells, where CPA1 expression is detected first, and elastase production is a later event (Fukayama et al., 1986; Jennings et al., 2013). Our observations of the expression patterns of pancreatic enzymes were also consistent with the morphology of secretory vesicles in acinar organoids, which resemble precursors but not the mature form of zymogen granules. About 30% of duct-like organoids expressed the mature duct marker CAII, consistent with the early stages of lineage specification. Consistent with these correlations, single-nucleus gene expression analysis identified similarities between our acinus-like and duct-like organoids and previously reported neonatal acinar and ductal signatures. We recognize that further optimization of culture conditions will be needed to induce generation of maturate of human acinar and ductal cells, which can aid in developing better models of PDAC. The results presented here, however, will help define conditions to induce ductal or acinar lineage commitment and identify a platform for understanding the mechanisms that regulate lineage commitment in the human pancreas.

We find that activating mutations in *KRAS* and *GNAS* show lineage tropism. *KRAS* was more effective in acinar cells, whereas *GNAS* was more effective in ductal epithelia. Although *KRAS^{G12D}* was competent in inducing proliferation in acinar and ductal lineages in culture, acinar cells were more effective than ductal cells in promoting formation of cancerous lesions *in vivo*. Consistent with the notion that IPMN originates from ductal epithelia (Ren et al., 2019), ductal acini responded strongly to *GNAS^{R201C}* expression by changes in morphology and expression of mucin (MUC2) associated with IPMN neoplastic lesions. Expression of *GNAS^{R201C}* in acinar cells failed to induce expression of MUC2, which is consistent with a previous study where acinus-specific expression of *Gnas^{R201C}* in mouse models was not sufficient to initiate PDAC (Patra et al., 2018). Understanding the mechanisms that regulate this lineage tropism and engineering genetic cooperation events to model initiation and progression of PDAC will provide important insights into events regulating initiation of pancreatic cancer.

We demonstrate that hPSCs can be induced to differentiate into ductal and acinar organoids representing the exocrine pancreas. In particular, our results identify a renewable source for generating human exocrine pancreas organoids for studying exocrine development and disease modeling.

Limitations of study

The organoids generated with our protocols represented fetal or neonatal exocrine pancreatic ducts and acini and did not represent adult lineages. We also lack data demonstrating the functions and growth of normal organoids *in vivo*. Further optimization of conditions may be needed to generate mature acinar and ductal organoids in culture that can grow *in vivo*. In addition, our single-nucleus analysis data did not have large cell numbers for ductal organoids, although the quality of the data was excellent. We analyzed cells 8 days after differentiation because day 8 was used for oncogene activation studies. It is possible that snRNA analysis of day 16 organoids would add strength to our data. Last, our studies of oncogene-induced cell state changes were performed in the cell population. Performing single-cell analysis will likely provide more insights into the biology of pancreatic cancer initiation. Although addressing the limitations can help increase the strength of our conclusions, the results presented already provide direct support for all conclusions drawn from this study.

STAR★METHODS

Detailed methods are provided in the online version of this paper and include the following:

- KEY RESOURCES TABLE
- RESOURCE AVAILABILITY
  - Lead contact
  - Materials availability
  - Data and code availability
- EXPERIMENTAL MODEL AND SUBJECT DETAILS
  - *In vivo* Animal Models
  - Pluripotent Stem Cell Lines
- METHOD DETAILS
  - Induction of Organoids
  - Phase Contrast Imaging and Analysis
  - Immunofluorescence
  - Western Blotting
  - Enzyme Function Analysis
  - RNA Extraction and qPCR Analysis
  - Virus production and Infection
  - Mouse Transplantation
  - Single nuclei RNA sequencing and analysis
- QUANTIFICATION AND STATISTICAL ANALYSIS

SUPPLEMENTAL INFORMATION

Supplemental information can be found online at https://doi.org/10.1016/j.stem.2021.03.022.

ACKNOWLEDGMENTS

We are grateful to members of the Melton laboratory, including Elise Engquist, Yi Yu, Kaleigh Biles, Jingping Zhang, and Kyle Boulanger, for technical support with stem cell culture and *in vitro* differentiation. We thank Dr. Anirban Maitra (MD Anderson) for providing wild-type and mutant GNAS cDNAs. We thank Dr. Christopher Wright (Vanderbilt University) for providing the PTF1A antibody. In addition, we thank members of the Muthuswamy laboratory for critical input throughout the development of this project. Institutional startup funds and UO1 (CA224193 to S.K.M.), an F32 fellowship (F32GM115201 to R.D.), seed grant from the Hirschberg Foundation for Pancreatic Cancer Research (to L.H.), and R01 from NIGMS (R01GM135462 to Z.G.) were used for completion of this study.

AUTHOR CONTRIBUTIONS

L.H. and S.K.M. conceived and designed the study, interpreted the results, and wrote the manuscript. L.H. developed the medium conditions for generation of acinar and ductal organoids and performed KRas experiments. R.D. performed all experiments using GNAS, analyzed the data, and wrote the corresponding results. C.M.L. and L.B.M. conducted bioinformatics analyses of TGCA pancreatic cancer cohorts. R.G. analyzed the histopathology of pancreatic transplants. D.N.C. and Z.G. performed single-nucleus gene expression studies, analyzed the data, and wrote the corresponding sections. N.C.L. generated PP lines from iPSCs.

DECLARATION OF INTERESTS

The authors declare no competing interests.

INCLUSION AND DIVERSITY

One or more of the authors of this paper self-identifies as an underrepresented ethnic minority in science. One or more of the authors of this paper self-identifies as a member of the LGBTQ+ community.

Received: April 30, 2020
Revised: February 14, 2021
Accepted: March 29, 2021
Published: April 28, 2021

REFERENCES

Aguirre, A.J., Bardeesy, N., Sinha, M., Lopez, L., Tuveson, D.A., Horner, J., Redston, M.S., and DePinho, R.A. (2003). Activated Kras and Ink4a/Arf deficiency cooperate to produce metastatic pancreatic ductal adenocarcinoma. Genes Dev. *17*, 3112–3126.

Aiello, N.M., Maddipati, R., Norgard, R.J., Balli, D., Li, J., Yuan, S., Yamazoe, T., Black, T., Sahmoud, A., Furth, E.E., et al. (2018). EMT Subtype Influences Epithelial Plasticity and Mode of Cell Migration. Dev. Cell *45*, 681–695.e4.

Almoguera, C., Shibata, D., Forrester, K., Martin, J., Arnheim, N., and Perucho, M. (1988). Most human carcinomas of the exocrine pancreas contain mutant c-K-ras genes. Cell *53*, 549–554.

Amato, E., Molin, M.D., Mafficini, A., Yu, J., Malleo, G., Rusev, B., Fassan, M., Antonello, D., Sadakari, Y., Castelli, P., et al. (2014). Targeted next-generation sequencing of cancer genes dissects the molecular profiles of intraductal papillary neoplasms of the pancreas. J. Pathol. *233*, 217–227.

Bardeesy, N., Aguirre, A.J., Chu, G.C., Cheng, K.H., Lopez, L.V., Hezel, A.F., Feng, B., Brennan, C., Weissleder, R., Mahmood, U., et al. (2006). Both p16(Ink4a) and the p19(Arf)-p53 pathway constrain progression of pancreatic adenocarcinoma in the mouse. Proc. Natl. Acad. Sci. USA *103*, 5947–5952.

Boj, S.F., Hwang, C.I., Baker, L.A., Chio, I.I., Engle, D.D., Corbo, V., Jager, M., Ponz-Sarvise, M., Tiriac, H., Spector, M.S., et al. (2015). Organoid models of human and mouse ductal pancreatic cancer. Cell *160*, 324–338.

Breunig, M., Merkle, J., Wagner, M., Melzer, M.K., Barth, T.F.E., Engleitner, T., Krumm, J., Wiedenmann, S., Cohrs, C.M., Perkhofer, L., et al. (2021). Modeling plasticity and dysplasia of pancreatic ductal organoids derived from human pluripotent stem cells. Cell Stem Cell *28*. Published online April 28, 2021. https://doi.org/10.1016/j.stem.2021.03.005.

Buscail, L., Bournet, B., and Cordelier, P. (2020). Role of oncogenic KRAS in the diagnosis, prognosis and treatment of pancreatic cancer. Nat. Rev. Gastroenterol. Hepatol. *17*, 153–168.

Collet, L., Ghurburrun, E., Meyers, N., Assi, M., Pirlot, B., Leclercq, I.A., Couvelard, A., Komuta, M., Cros, J., Demetter, P., et al. (2020). *Kras* and *Lkb1* mutations synergistically induce intraductal papillary mucinous neoplasm derived from pancreatic duct cells. Gut *69*, 704–714.

Cooper, C.L., O'Toole, S.A., and Kench, J.G. (2013). Classification, morphology and molecular pathology of premalignant lesions of the pancreas. Pathology *45*, 286–304.

De La O, J.P., Emerson, L.L., Goodman, J.L., Froebe, S.C., Illum, B.E., Curtis, A.B., and Murtaugh, L.C. (2008). Notch and Kras reprogram pancreatic acinar cells to ductal intraepithelial neoplasia. Proc. Natl. Acad. Sci. USA *105*, 18907–18912.

De Lisle, R.C., and Logsdon, C.D. (1990). Pancreatic acinar cells in culture: expression of acinar and ductal antigens in a growth-related manner. Eur. J. Cell Biol. *51*, 64–75.

Dekkers, J.F., Wiegerinck, C.L., de Jonge, H.R., Bronsveld, I., Janssens, H.M., de Winter-de Groot, K.M., Brandsma, A.M., de Jong, N.W., Bijvelds, M.J., Scholte, B.J., et al. (2013). A functional CFTR assay using primary cystic fibrosis intestinal organoids. Nat. Med. *19*, 939–945.

Esni, F., Ghosh, B., Biankin, A.V., Lin, J.W., Albert, M.A., Yu, X., MacDonald, R.J., Civin, C.I., Real, F.X., Pack, M.A., et al. (2004). Notch inhibits Ptf1 function and acinar cell differentiation in developing mouse and zebrafish pancreas. Development *131*, 4213–4224.

Ferreira, R.M.M., Sancho, R., Messal, H.A., Nye, E., Spencer-Dene, B., Stone, R.K., Stamp, G., Rosewell, I., Quaglia, A., and Behrens, A. (2017). Duct- and Acinar-Derived Pancreatic Ductal Adenocarcinomas Show Distinct Tumor Progression and Marker Expression. Cell Rep. *21*, 966–978.

Fukayama, M., Ogawa, M., Hayashi, Y., and Koike, M. (1986). Development of human pancreas. Immunohistochemical study of fetal pancreatic secretory proteins. Differentiation *31*, 127–133.

Gidekel Friedlander, S.Y., Chu, G.C., Snyder, E.L., Girnius, N., Dibelius, G., Crowley, D., Vasile, E., DePinho, R.A., and Jacks, T. (2009). Context-dependent transformation of adult pancreatic cells by oncogenic K-Ras. Cancer Cell *16*, 379–389.

Gillies, T.E., Pargett, M., Silva, J.M., Teragawa, C.K., McCormick, F., and Albeck, J.G. (2020). Oncogenic mutant RAS signaling activity is rescaled by the ERK/MAPK pathway. Mol. Syst. Biol. *16*, e9518.

Guerra, C., Schuhmacher, A.J., Cañamero, M., Grippo, P.J., Verdaguer, L., Pérez-Gallego, L., Dubus, P., Sandgren, E.P., and Barbacid, M. (2007). Chronic pancreatitis is essential for induction of pancreatic ductal adenocarcinoma by K-Ras oncogenes in adult mice. Cancer Cell *11*, 291–302.

Habbe, N., Shi, G., Meguid, R.A., Fendrich, V., Esni, F., Chen, H., Feldmann, G., Stoffers, D.A., Konieczny, S.F., Leach, S.D., and Maitra, A. (2008). Spontaneous induction of murine pancreatic intraepithelial neoplasia (mPanIN) by acinar cell targeting of oncogenic Kras in adult mice. Proc. Natl. Acad. Sci. USA *105*, 18913–18918.

Hafemeister, C., and Satija, R. (2019). Normalization and variance stabilization of single-cell RNA-seq data using regularized negative binomial regression. Genome Biol. *20*, 296.

Handler, J., Cullis, J., Avanzi, A., Vucic, E.A., and Bar-Sagi, D. (2018). Pre-neoplastic pancreas cells enter a partially mesenchymal state following transient TGF-β exposure. Oncogene *37*, 4334–4342.

Haumaitre, C., Lenoir, O., and Scharfmann, R. (2008). Histone deacetylase inhibitors modify pancreatic cell fate determination and amplify endocrine progenitors. Mol. Cell. Biol. *28*, 6373–6383.

Hingorani, S.R., Petricoin, E.F., Maitra, A., Rajapakse, V., King, C., Jacobetz, M.A., Ross, S., Conrads, T.P., Veenstra, T.D., Hitt, B.A., et al. (2003). Preinvasive and invasive ductal pancreatic cancer and its early detection in the mouse. Cancer Cell *4*, 437–450.

Hohwieler, M., Illing, A., Hermann, P.C., Mayer, T., Stockmann, M., Perkhofer, L., Eiseler, T., Antony, J.S., Müller, M., Renz, S., et al. (2017). Human pluripotent stem cell-derived acinar/ductal organoids generate human pancreas upon orthotopic transplantation and allow disease modelling. Gut *66*, 473–486.

Hruban, R.H., Goggins, M., Parsons, J., and Kern, S.E. (2000). Progression model for pancreatic cancer. Clin. Cancer Res. *6*, 2969–2972.

Hruban, R.H., Maitra, A., Kern, S.E., and Goggins, M. (2007). Precursors to pancreatic cancer. Gastroenterol. Clin. North Am. *36*, 831–849, vi.

Huang, L., Holtzinger, A., Jagan, I., BeGora, M., Lohse, I., Ngai, N., Nostro, C., Wang, R., Muthuswamy, L.B., Crawford, H.C., et al. (2015). Ductal pancreatic cancer modeling and drug screening using human pluripotent stem cell- and patient-derived tumor organoids. Nat. Med. *21*, 1364–1371.

Huch, M., Bonfanti, P., Boj, S.F., Sato, T., Loomans, C.J., van de Wetering, M., Sojoodi, M., Li, V.S., Schuijers, J., Gracanin, A., et al. (2013). Unlimited in vitro expansion of adult bi-potent pancreas progenitors through the Lgr5/R-spondin axis. EMBO J. *32*, 2708–2721.

Ideno, N., Yamaguchi, H., Ghosh, B., Gupta, S., Okumura, T., Steffen, D.J., Fisher, C.G., Wood, L.D., Singhi, A.D., Nakamura, M., et al. (2018). GNAS(R201C) Induces Pancreatic Cystic Neoplasms in Mice That Express Activated KRAS by Inhibiting YAP1 Signaling. Gastroenterology *155*, 1593–1607.e12.

Jennings, R.E., Berry, A.A., Kirkwood-Wilson, R., Roberts, N.A., Hearn, T., Salisbury, R.J., Blaylock, J., Piper Hanley, K., and Hanley, N.A. (2013). Development of the human pancreas from foregut to endocrine commitment. Diabetes *62*, 3514–3522.

Kanda, M., Matthaei, H., Wu, J., Hong, S.M., Yu, J., Borges, M., Hruban, R.H., Maitra, A., Kinzler, K., Vogelstein, B., and Goggins, M. (2012). Presence of somatic mutations in most early-stage pancreatic intraepithelial neoplasia. Gastroenterology *142*, 730–733.e9.

Keefe, M.D., Wang, H., De La O, J.P., Khan, A., Firpo, M.A., and Murtaugh, L.C. (2012). β-catenin is selectively required for the expansion and regeneration of mature pancreatic acinar cells in mice. Dis. Model. Mech. *5*, 503–514.

Kim, G.E., Bae, H.I., Park, H.U., Kuan, S.F., Crawley, S.C., Ho, J.J., and Kim, Y.S. (2002). Aberrant expression of MUC5AC and MUC6 gastric mucins and sialyl Tn antigen in intraepithelial neoplasms of the pancreas. Gastroenterology *123*, 1052–1060.

Kleeff, J., Korc, M., Apte, M., La Vecchia, C., Johnson, C.D., Biankin, A.V., Neale, R.E., Tempero, M., Tuveson, D.A., Hruban, R.H., and Neoptolemos, J.P. (2016). Pancreatic cancer. Nat. Rev. Dis. Primers *2*, 16022.

Kopp, J.L., Dubois, C.L., Schaeffer, D.F., Samani, A., Taghizadeh, F., Cowan, R.W., Rhim, A.D., Stiles, B.L., Valasek, M., and Sander, M. (2018). Loss of Pten and Activation of Kras Synergistically Induce Formation of Intraductal Papillary Mucinous Neoplasia From Pancreatic Ductal Cells in Mice. Gastroenterology *154*, 1509–1523.e5.

Kopp, J.L., von Figura, G., Mayes, E., Liu, F., Dubois, C.L., Morris, J.P., IV, Pan, F.C., Akiyama, H., Wright, C.V.E., Jensen, K., Hebrok, M., and Sander, M. (2012). Identification of Sox9-dependent acinar-to-ductal reprogramming as the principal mechanism for initiation of pancreatic ductal adenocarcinoma. Cancer Cell 22 (6), 737–750, https://doi.org/10.1016/j.ccr.2012.10.025.

Laitio, M., Lev, R., and Orlic, D. (1974). The developing human fetal pancreas: an ultrastructural and histochemical study with special reference to exocrine cells. J. Anat. *117*, 619–634.

Lee, J., Snyder, E.R., Liu, Y., Gu, X., Wang, J., Flowers, B.M., Kim, Y.J., Park, S., Szot, G.L., Hruban, R.H., et al. (2017). Reconstituting development of pancreatic intraepithelial neoplasia from primary human pancreas duct cells. Nat. Commun. *8*, 14686.

Lee, A.Y.L., Dubois, C.L., Sarai, K., Zarei, S., Schaeffer, D.F., Sander, M., and Kopp, J.L. (2019). Cell of origin affects tumour development and phenotype in pancreatic ductal adenocarcinoma. Gut *68*, 487–498.

Leite, N.C., Sintov, E., Meissner, T.B., Brehm, M.A., Greiner, D.L., Harlan, D.M., and Melton, D.A. (2020). Modeling Type 1 Diabetes In Vitro Using Human Pluripotent Stem Cells. Cell Rep. *32*, 107894.

Liu, J., Akanuma, N., Liu, C., Naji, A., Halff, G.A., Washburn, W.K., Sun, L., and Wang, P. (2016). TGF-β1 promotes acinar to ductal metaplasia of human pancreatic acinar cells. Sci. Rep. *6*, 30904.

Lugea, A., Waldron, R.T., Mareninova, O.A., Shalbueva, N., Deng, N., Su, H.Y., Thomas, D.D., Jones, E.K., Messenger, S.W., Yang, J., et al. (2017). Human Pancreatic Acinar Cells: Proteomic Characterization, Physiologic Responses, and Organellar Disorders in ex Vivo Pancreatitis. Am. J. Pathol. *187*, 2726–2743.

Maitra, A., and Hruban, R.H. (2008). Pancreatic cancer. Annu. Rev. Pathol. *3*, 157–188.

Masui, T., Swift, G.H., Deering, T., Shen, C., Coats, W.S., Long, Q., Elsässer, H.P., Magnuson, M.A., and MacDonald, R.J. (2010). Replacement of Rbpj with Rbpjl in the PTF1 complex controls the final maturation of pancreatic acinar cells. Gastroenterology *139*, 270–280.

McGinnis, C.S., Patterson, D.M., Winkler, J., Conrad, D.N., Hein, M.Y., Srivastava, V., Hu, J.L., Murrow, L.M., Weissman, J.S., Werb, Z., et al. (2019). MULTI-seq: sample multiplexing for single-cell RNA sequencing using lipid-tagged indices. Nat. Methods *16*, 619–626.

McQuin, C., Goodman, A., Chernyshev, V., Kamentsky, L., Cimini, B.A., Karhohs, K.W., Doan, M., Ding, L., Rafelski, S.M., Thirstrup, D., et al. (2018). CellProfiler 3.0: Next-generation image processing for biology. PLoS Biol. *16*, e2005970.

Mueller, S., Engleitner, T., Maresch, R., Zukowska, M., Lange, S., Kaltenbacher, T., Konukiewitz, B., Öllinger, R., Zwiebel, M., Strong, A., et al. (2018). Evolutionary routes and KRAS dosage define pancreatic cancer phenotypes. Nature *554*, 62–68.

Murtaugh, L.C., Stanger, B.Z., Kwan, K.M., and Melton, D.A. (2003). Notch signaling controls multiple steps of pancreatic differentiation. Proc. Natl. Acad. Sci. USA *100*, 14920–14925.

Ohtsuka, T., Tomosugi, T., Kimura, R., Nakamura, S., Miyasaka, Y., Nakata, K., Mori, Y., Morita, M., Torata, N., Shindo, K., et al. (2019). Clinical assessment of the GNAS mutation status in patients with intraductal papillary mucinous neoplasm of the pancreas. Surg. Today *49*, 887–893.

Pagliuca, F.W., Millman, J.R., Gürtler, M., Segel, M., Van Dervort, A., Ryu, J.H., Peterson, Q.P., Greiner, D., and Melton, D.A. (2014). Generation of functional human pancreatic β cells in vitro. Cell *159*, 428–439.

Patra, K.C., Kato, Y., Mizukami, Y., Widholz, S., Boukhali, M., Revenco, I., Grossman, E.A., Ji, F., Sadreyev, R.I., Liss, A.S., et al. (2018). Mutant GNAS drives pancreatic tumourigenesis by inducing PKA-mediated SIK suppression and reprogramming lipid metabolism. Nat. Cell Biol. *20*, 811–822.

Reichert, M., and Rustgi, A.K. (2011). Pancreatic ductal cells in development, regeneration, and neoplasia. J. Clin. Invest. *121*, 4572–4578.

Ren, B., Liu, X., and Suriawinata, A.A. (2019). Pancreatic Ductal Adenocarcinoma and Its Precursor Lesions: Histopathology, Cytopathology, and Molecular Pathology. Am. J. Pathol. *189*, 9–21.

Riesle, E., Friess, H., Zhao, L., Wagner, M., Uhl, W., Baczako, K., Gold, L.I., Korc, M., and Büchler, M.W. (1997). Increased expression of transforming growth factor beta s after acute oedematous pancreatitis in rats suggests a role in pancreatic repair. Gut *40*, 73–79.

Rooman, I., Heremans, Y., Heimberg, H., and Bouwens, L. (2000). Modulation of rat pancreatic acinoductal transdifferentiation and expression of PDX-1 in vitro. Diabetologia *43*, 907–914.

Rovira, M., Delaspre, F., Massumi, M., Serra, S.A., Valverde, M.A., Lloreta, J., Dufresne, M., Payre, B., Konieczny, S.F., Savatier, P., et al. (2008). Murine embryonic stem cell-derived pancreatic acinar cells recapitulate features of early pancreatic differentiation. Gastroenterology *135*, 1301–1310, 1310.e1–5.

Seino, T., Kawasaki, S., Shimokawa, M., Tamagawa, H., Toshimitsu, K., Fujii, M., Ohta, Y., Matano, M., Nanki, K., Kawasaki, K., et al. (2018). Human Pancreatic Tumor Organoids Reveal Loss of Stem Cell Niche Factor Dependence during Disease Progression. Cell Stem Cell *22*, 454–467.e6.

Serrano, M., Lin, A.W., McCurrach, M.E., Beach, D., and Lowe, S.W. (1997). Oncogenic ras provokes premature cell senescence associated with accumulation of p53 and p16INK4a. Cell *88*, 593–602.

Sharma, A., Sances, S., Workman, M.J., and Svendsen, C.N. (2020). Multi-lineage Human iPSC-Derived Platforms for Disease Modeling and Drug Discovery. Cell Stem Cell *26*, 309–329.

Shih, H.P., Kopp, J.L., Sandhu, M., Dubois, C.L., Seymour, P.A., Grapin-Botton, A., and Sander, M. (2012). A Notch-dependent molecular circuitry initiates pancreatic endocrine and ductal cell differentiation. Development *139*, 2488–2499.

Simsek, S., Zhou, T., Robinson, C.L., Tsai, S.Y., Crespo, M., Amin, S., Lin, X., Hon, J., Evans, T., and Chen, S. (2016). Modeling Cystic Fibrosis Using Pluripotent Stem Cell-Derived Human Pancreatic Ductal Epithelial Cells. Stem Cells Transl. Med. *5*, 572–579.

Stuart, T., Butler, A., Hoffman, P., Hafemeister, C., Papalexi, E., Mauck, W.M., 3rd, Hao, Y., Stoeckius, M., Smibert, P., and Satija, R. (2019). Comprehensive Integration of Single-Cell Data. Cell *177*, 1888–1902.e21.

Taki, K., Ohmuraya, M., Tanji, E., Komatsu, H., Hashimoto, D., Semba, K., Araki, K., Kawaguchi, Y., Baba, H., and Furukawa, T. (2016). GNAS(R201H) and Kras(G12D) cooperate to promote murine pancreatic tumorigenesis recapitulating human intraductal papillary mucinous neoplasm. Oncogene *35*, 2407–2412.

Tosti, L., Hang, Y., Debnath, O., Tiesmeyer, S., Trefzer, T., Steiger, K., Ten, F.W., Lukassen, S., Ballke, S., Kuhl, A.A., et al. (2021). Single-Nucleus and In Situ RNA-Sequencing Reveal Cell Topographies in the Human Pancreas. Gastroenterology *160*, 1330–1334.e11.

Tuveson, D.A., Shaw, A.T., Willis, N.A., Silver, D.P., Jackson, E.L., Chang, S., Mercer, K.L., Grochow, R., Hock, H., Crowley, D., et al. (2004). Endogenous oncogenic K-ras(G12D) stimulates proliferation and widespread neoplastic and developmental defects. Cancer Cell *5*, 375–387.

Tuveson, D.A., Zhu, L., Gopinathan, A., Willis, N.A., Kachatrian, L., Grochow, R., Pin, C.L., Mitin, N.Y., Taparowsky, E.J., Gimotty, P.A., et al. (2006). Mist1-KrasG12D knock-in mice develop mixed differentiation metastatic exocrine pancreatic carcinoma and hepatocellular carcinoma. Cancer Res. *66*, 242–247.

Uhlén, M., Fagerberg, L., Hallström, B.M., Lindskog, C., Oksvold, P., Mardinoglu, A., Sivertsson, Å., Kampf, C., Sjöstedt, E., Asplund, A., et al. (2015). Proteomics. Tissue-based map of the human proteome. Science *347*, 1260419.

Waters, A.M., and Der, C.J. (2018). KRAS: The Critical Driver and Therapeutic Target for Pancreatic Cancer. Cold Spring Harb. Perspect. Med. *8*, a031435.

Wu, J., Matthaei, H., Maitra, A., Dal Molin, M., Wood, L.D., Eshleman, J.R., Goggins, M., Canto, M.I., Schulick, R.D., Edil, B.H., et al. (2011). Recurrent GNAS mutations define an unexpected pathway for pancreatic cyst development. Sci. Transl. Med. *3*, 92ra66.

Xu, J., Lamouille, S., and Derynck, R. (2009). TGF-beta-induced epithelial to mesenchymal transition. Cell Res. *19*, 156–172.

STAR★METHODS

KEY RESOURCES TABLE

REAGENTS or RESOURCE	SOURCE	IDENTIFIER
Antibodies		
SOX9	Millipore	AB5535
CAII	Santa Cruz	sc-133111
PTF1A	Christopher Wright Lab (Vanderbilt)	N/A
PTF1A	BD Biosciences	s25-763
CTRC	Sino Biological	11456-T24
CPA1	Sino Biological	10504-RP02
MUC2	Santa Cruz	sc7314
MUC5AC	Santa Cruz	sc33667
phospho-ERK	Cell Signaling	4370
KRAS	Cell Signaling	3339
ERK	Cell Signaling	9107
KRT19	Abcam	ab9221
Ki67	Cell Signaling	9027
KRT7	DAKO	M7018
P16	Santa Cruz	sc-1661
Goat anti-mouse IgG	Invitrogen	A-21424
Goat anti-rabbit IgG	Invitrogen	A-11034
Oligonucleotides		
TBP (PCR primers)	IDT	Hs.PT.39a.22214825
PDX1 (PCR primers)	IDT	Hs.PT.58.2295117
NXK6-1 (PCR primers)	IDT	Hs.PT.58.25073618
GATA4 (PCR primers)	IDT	Hs.PT.58.259457
SOX9 (PCR primers)	IDT	Hs.PT.58.38984663
CFTR (PCR primers)	IDT	Hs.PT.58.3365414
PTF1A (PCR primers)	IDT	Hs.PT.58.45382933.g
CPA1 (PCR primers)	IDT	Hs.PT.58.3138313
CTRC (PCR primers)	IDT	Hs.PT.58.15671909
HNF1B (PCR primers)	IDT	Hs.PT.58.25568705
RBBPJL (PCR primers)	IDT	Hs.PT.58.15167632.gs
Critical commercial assays		
Amylase Activity Colorimetric Assay Kit	Biovision	K711
Lipase Activity Fluorometric Assay Kit III	Biovision	K724
Anhydrase (CA) Activity Assay Kit	Biovision	K472
Alcian Blue (pH 2.5) Stain Kit	Vector Laboratories	H3501
Deposited data		
Single nuclei transcriptome	GEO repository	GEO: GSE169008
Experimental models: cell lines		
HUES8	Douglas Metlon Lab (Harvard University)	CVCL_B207
iPSC #11	Douglas Metlon Lab (Harvard University)	N/A
iPSC #13	Douglas Metlon Lab (Harvard University)	N/A
Experimental models: organisms		
NCG mice	Charles River	572

(*Continued on next page*)

Continued		
REAGENTS or RESOURCE	SOURCE	IDENTIFIER
Recombinant DNA		
pINDUCER21 (ORF-EG)	Addgene	#46948
Biological samples		
PDAC patient tissue #1 (paraffin slide)	BIDMC	N/A
PDAC patient tissue #2 (paraffin slide)	BIDMC	N/A
Software and algorithms		
Analysis Code	Github	https://github.com/dannyconrad/PancreasOrganoidMULTIseq
Chemicals, peptides, and recombinant proteins		
Forskolin	Selleckchem	S2449
BSA (fatty acid-free)	Proliant Biologicals	68700
PBS	Invitrogen	14155063
DEMEM	Invitrogen	10564045
TrypLE	Invitrogen	12605010
Matrigel	Corning	354263
Cell Recovery Solution	Corning	354253
Trizol	Invitrogen	15596018
Direct-zol	Zymo Research	R2080
Superscript IV	Invitrogen	11756500
STEMxyme I	Worthington Biochemical	STZ1
Doxycycline diet	Envigo	TD01306
TGF	Peprotech	100-21
FGF10	Peprotech	100-26
FGF2	Peprotech	100-18C
EGF	Peprotech	AF-100-15
Doxycycline (powder)	Tocris	4090
A83-01	Tocris	2939
Y27632	Tocris	1245
SKL2001	Selleckchem	S8320
WNT1	Peprotech	120-17
Foxy5	Tocris	5461
Dexamethasone	Tocris	1126
LDN193189	Tocris	6053
CD3254	Tocris	3302
BMS961	Tocris	3410
DBZ	Tocris	4489
HPI-1	Tocris	3839
XMU-MP-1	Selleckchem	S8334
CHIR99021	Tocris	4953
Ascorbic acid	Sigma	A4544
EPZ011989	Selleckchem	S7805
IWP2	Tocris	3533
IQ1	Tocris	4713
iCRT-14	Tocris	4299
CPTH2	Cayman Chemical	12086
SB939	Selleckchem	S1515
WT161	Selleckchem	S8495
XAV939	Tocris	3748
FGF1	Peprotech	100-17A

CellPress
OPEN ACCESS

RESOURCE AVAILABILITY

Lead contact

Further information and requests for resources and reagents should be directed and will be fulfilled by the lead contact, Senthil K Muthuswamy (smuthusw@bidmc.harvard.edu)

Materials availability

This study did not generate new unique reagents.

Data and code availability

The single nucleus transcriptome data generated in this study will be available at GEO repository with accession number GEO: GSE169008. R language code for analysis will be available at https://github.com/dannyconrad/PancreasOrganoidMULTIseq.

EXPERIMENTAL MODEL AND SUBJECT DETAILS

In vivo Animal Models

Animal experiments performed in this study followed protocols approved by the institutional animal care and use committee at Beth Israel Deaconess Medical Center (Boston, USA). Male immunodeficient NOD CRISPR *Prkdc Il2r Gamma* (NCG) mice of 6-8 week old were purchased from Charles River (catalog #572). Results for each experimental group are summary of two to three independent operations using independent organoid cultures.

Pluripotent Stem Cell Lines

Human embryonic stem line HUES8 was generated at Harvard University and has a normal 46 XY karyotype (NIH registration number 0021; RRID: CVCL_B207). Induced pluripotent stem cell lines 11 and 13 were generated by the Melton laboratory at Harvard University. Donor for line 11 is a 26 year old healthy male. Donor for line 13 is a 27 year old healthy female. pINDUCER21 (Addgene #46948) was used for transgene expression.

METHOD DETAILS

Induction of Organoids

Pancreatic progenitor cells were derived from Hues-8 cells by Dr. Douglas Melton's group, following a previously published protocol (Leite et al., 2020; Pagliuca et al., 2014). The PP1 cell aggregates at stage S4+2d induction was collected and dissociated with TrypLE (Invitrogen) into single cells. Digested cells were centrifuged at 1500 rpm for 5 min then resuspended in differentiation media supplemented with 5% Matrigel, at final cell density of 50,000/ml. Cell suspensions were seeded in culture plates or chamber slides precoated with 100% Matrigel. Media were replaced every four days, all supplemented with 5% Matrigel. For ductal cell induction, the basic medium contains DMEM, 1% Pen-Strep, 1% B27, 10 ng/ml FGF1, 50 μg/ml ascorbic acid, 1 μM A83-01, 10 ng/ml FGF10, 1 ng/ml EGF, 100 nM BMS961. Stage I duct medium (day 0-4) contains basic medium, supplemented with 15 ng FGF10, 10 ng/ml FGF2, 5 ng/ml EGF, 100 nM EPZ011989, 1 μg/ml Foxy5, 3 μM IWP2, 25 nM IQ1, 50 nM iCRT-14, 10 μM Y27632, 1 μM CPTH2, 50 nM SB939. Stage II (day 4-8) duct medium contains basic medium, supplemented with 1 μg/ml Foxy5, 3 μM IWP2, 25 nM IQ1, 50 nM iCRT-14, 50 nM WT161, 5 μM Y27632, 5 μM CPTH2. Stage III (day 8-12) duct medium contains basic medium, supplemented with 3μM IWP2, 25 nM IQ1, 50 nM iCRT-14, 5 μM CPTH2, 400 nM BMS961, 0.1 μM XAV939. Stage IV (day 12-) duct medium contains basic medium, supplemented with 3 μM IWP2, 5 nM IQ1, 10 nM iCRT-14, 0.5 μM CPTH2. For acinar induction, the basic medium contains DMEM, 1% Pen-Strep, 1% B27, 10 ng/ml FGF1, 50 μg/ml ascorbic acid, 1 μM A83-01, 10 ng/ml WNT1. Stage I acinar (day 0-4) medium contains basic medium, supplemented with 10 μM Y27632, 3μM SKL2001, 15 ng/ml WNT1, 50 nM dexamethasone, 5 ng/ml FGF2, 1 ng/EGF, 0.3 μM CHIR, 5 ng/ml FGF10, 0.1 μM HPI-1, 50 nM XMU-MP-1. Stage II (day 4-8) acinar medium contains basic medium, supplemented with 3 μM SKL2001, 200 nM dexamethasone, 0.1 μM HPI-1, LDN193189 0.1 μM, 5 nM CD3254, 50 nM XMU-MP-1. Stage III (day8-12) acinar medium contains basic medium, supplemented 3 μM SKL2001, 200 nM dexamethasone, 0.1 μM LDN193189, 1 μM DBZ. Stage IV (day12-) acinar medium contains basic medium, 0.3 μM SKL2001, 25 nM dexamethasone, 0.1 μM DBZ. For TGFβ treatment experiments, A83-01 were removed from medium from day 8.

Phase Contrast Imaging and Analysis

For phase contrast imaging, organoids were seeded in Falcon culture chamber slide and images were taken every two days using 10X objective. For each time point, three independent cultures were imaged with at least 100 organoids imaged per culture. For analysis of phase contrast images, CellProfiler program (McQuin et al., 2018) was used to measure the Area and Form Factor. Form Factor is defined as $4*\pi*Area/Perimeter^2$.

Immunofluorescence

For direct immunostaining, organoids were seeded in Falcon culture chamber slides. At the time of analysis, organoids in chambers were washed once with PBS then fixed with 4% PFA for 40 minutes. After fixation, organoids were washed three times with

PBS-glycine (100mM glycine) with 10 minute incubation each wash. Organoids then were permeabilized by 0.5% Triton X-100 of 5 minute incubation. After permeabilization, organoids were blocked using blocking buffer (PBS, 100 mM glycine, 0.1% BSA, 1% goat serum, 0.05% Triton X-100) for one hour. For primary antibody staining, antibodies were diluted with the blocking buffer and organoids were incubated with the dilution for 2-4 hours at room temperature then washed three times with blocking buffer (10 minutes incubation per wash). For secondary antibody staining, goat anti-mouse IgG or goat anti-rabbit IgG antibodies from Invitrogen were diluted at 1:400 in blocking buffer then incubated organoids with the dilution for 1.5 hour. After the secondary staining, organoids were washed with PBS three times and stained with DAPI for 5 minute. Slides were mounted with DAKO fluorescence mounting media. Alcian blue staining were performed using the Alcian Blue (pH2.5) Stain Kit (#H3501, Vector Laboratories). Imaging of slides were conducted under Zeiss LSM880 confocal system, using 20X objective or a Keyence BZ microscope. Image analyses were performed using CellProfiler.

For preparation of paraffin blocks, organoids in chamber slides were fixed with 4% PFA for 2 hours then embedded with Histogel in cryomold. Histogel capsules were transferred into cassette and processed at BIDMC histology core using standard paraffin embedding protocols. For staining of slides, paraffine sections of 5 μm thick were incubated with xylene 2x5 minutes then rehydrated in 100%, 95%, 75% ethanol and H_2O for 10 minute per incubation. Antigen retrieval were done by incubating slides with citrus buffer at high pressure heating for 15 minutes. After this, slides were washed twice with PBS then blocked with blocking buffer for one hour. Primary staining and secondary staining were done by incubating slides with antibodies for one hour at room temperature, with three washes in between using blocking buffer. After secondary staining, slides were washed with PBS three times and stained with DAPI for 5 minutes. Slides were mounted with DAKO fluorescence mounting media. Slides were imaged using Zeiss LSM or Keyence Vision System. Antibodies used: SOX9, 1:200, #AB5535, Millipore ; CAII, 1:100, #sc-133111, Santa Cruz; PTF1A, 1:100, #s25-763, BD ; CTRC, 1:100, #11456-T24, Sino Biological; CPA1,1:100, #10504-RP02, Sino Biological; Muc2, 1:100, #sc7314, Santa Cruz; Muc5AC, 1:100, #sc33667, Santa Cruz; phosphor-ERK, 1:200, #4370, Cell Signaling; KRT19, 1:200, #ab9221, Abcam;

Western Blotting

For western blotting, organoids were seeded in 6 well plates. At the time of analysis, culture media were aspirated then 1ml ice-cold PBS was added into one well. Pipette several times to physically disrupt the Matrigel and transfer the slurry into 15ml conical tube. Centrifuge tubes at 3500 rpm x 5minutes. Aspirate the supernatant carefully to not disrupt Matrigel layers. Add 1ml of ice-cold Cell Recovery Solution (Corning) and pipette several times then incubated tubes on ice for 1 hour. After the incubation, centrifuge tubes at 3500rpm x 5 minutes and aspirate the supernatant carefully not to disrupt cell pellets. Add 1ml of PBS to wash the pellet then centrifuge tubes at 3500rpm x 5 minutes and aspirate the supernatant carefully not to disrupt cell pellets. Pellets were lysed with RIPA buffer supplemented with protease and phosphatase inhibitors (Roche). Protein concentration of cell lysates were determined by Bradford assay and lysates were subject to standard procedures of western blotting. Antibodies use: Sox9, 1:500, #AB5535, Millipore; CAII, 1:500, #sc-133111, Santa Cruz; PTF1A, 1:500, a gift from Dr. Christopher Wright's lab (Vanderbilt University); CTRC, 1:500, #11456-T24, Sino Biological.

Enzyme Function Analysis

Organoids were seeded in 6 well plate. At the time of analysis, culture media were aspirated then 1ml ice-cold PBS was added into one well. Pipette several times to physically disrupt the Matrigel and transfer the slurry into 15ml conical tube. Centrifuge tubes at 3500 rpm x 5minutes. Aspirate the supernatant carefully to not disrupt Matrigel layers. Add 1ml of ice-cold Cell Recovery Solution (Corning) and pipette several times then incubated tubes on ice for 1 hour. After the incubation, centrifuge tubes at 3500rpm x 5 minutes and aspirate the supernatant carefully not to disrupt cell pellets. Add 1ml of PBS to wash the pellet then centrifuge tubes at 3500rpm x 5 minutes and aspirate the supernatant carefully not to disrupt cell pellets. Pellets were lysed with enzyme analysis buffer (150 mM NaCl, 10 mM Tris, 1% Triton X-100, pH = 7.2) with no protease inhibitors supplemented. The protein concentration of cell lysates was determined by Bradford assay. Enzyme activities were analyzed using Lipase Activity Fluorometric Assay Kit (#K724, Biovision) and Carbonic Anhydrase Activity Assay Kit (#K472, Biovision), following the instructions by the manufacturer.

RNA Extraction and qPCR Analysis

Organoids were seeded in 12 well plates. At the time of analysis, culture media were aspirated and 0.5 mL of ice-cold Cell Recovery Solution (Corning) was added into one well. Pipette several times to physically disrupt the Matrigel and transfer the slurry into 1.5ml microcentrifuge tube and incubate on ice. After 30 minutes, centrifuge the tubes at 8000rpm x 5 minutes. Aspirate supernatant then add 250 ul Trizol (Invitrogen). Lyze pellets for 15 minutes at room temperature. Purification of total RNA was done using Direct-Zol RNA kits (ZymoResearch). cDNA was synthesized using SuperScript IV First-Strand Master kit (Invitrogen). Gene expression was quantitated by quantitative PCR using PrimeTime qPCR Probe Assays kit.

Virus production and Infection

Mutant genes were cloned into pINDUCER21 (Addgene #46948) and lentivirues produced were concentrated by ultracentrifugaton at 25,000rpm for 2.5 hours at 4 degrees. Viruses were resuspended in PBS. 6-well plate was coated with Matrigel by incubating with 5% Matrigel for 1 hour then aspirate the solution to air dry. 500,000 pancreatic progenitor cells with culture media were seeded in one well and allowed to grow overnight. On second day, lentiviruses were mixed with culture media supplemented with 4ug/ml polybrene and 2ml solution was added into each well. To improve infection rate, plates were centrifuged at 3500rpm for 30 mins. The plates were

placed in incubator overnight. On third day, cells were digested with Accutase (Sigma), collected and seeded in 3D. For induction of transgene, doxycycline was added into culture media at final concentration of 1ug/ml.

Mouse Transplantation

Day 16 organoid cultures with or without $KRas^{G12D}$ expression were digested with STEMxyme I (1mg/ml) for 1.5 hours then dissociated with Accutase (Sigma). Dissociated cells were counted and resuspended in 90% Matrigel with a final density of 10 million cells/ml. Orthotopic mouse pancreas transplantation was conducted following the protocol approved by the institutional animal care and use committee at BIDMC. 6-8 week old male NCG mice (#572, Charles River) were purchased for this study. Mice were anesthetized by isoflurane and a 1.5 cm incision was cut at the left upper abdominal flank. The pancreas was carefully pulled out, and 50 ul of cell suspension was injected at the tail of the pancreas using a 29G needle. After injection, the pancreas was gently placed back into the peritoneal cavity, and the wound was sutured. Meloxicam was used as analgesics. Mice were fed with regular food or diets with doxycycline (#TD01306, Envigo). Eight weeks after injection, mice were sacrificed following approved protocol, and the pancreas was collected then processed as paraffin blocks.

For mammary fat pad transplantation, experiments were performed following the protocol approved by the institutional animal care and use committee at BIDMC. 6-8 week old female NCG mice (#572, Charles River) were purchased for this study. At the time of operation, Mice were anesthetized by isoflurane and a 1.5 cm incision was cut on the skin near No.9 and No.4 glands. A 29G needle was used to inject 50ul of cell/Matrigel suspension into mammary fat pad, at density of 500,000 cells/gland. After injection, the wound was sutured and Meloxicam was used as analgesics. Mice were fed with regular food or diets with doxycycline (#TD01306, Envigo). Eight weeks after injection, mice were sacrificed following approved protocol and pancreas was collected then processed as paraffin blocks.

Single nuclei RNA sequencing and analysis

Isolation of single nuclei from organoids were performed based on a published protocol (Tosti et al., 2021). 2ml of citric acid-based buffer (sucrose 0.25 M, citric acid 25 mM, $MgCl_2$ 3mM) were added to one well of 6-well plate with organoid culture. Slurries were collected then centrifuged at 800 g for 3 minutes at 4 degrees. Supernatant was discarded and cell pellets were incubated with 2 mL of citric acid-Triton buffer (sucrose 0.25 M, citric acid 25 mM, $MgCl_2$ 3mM, 0.05% Triton X-100) for 3 minutes on ice then centrifuged at 800 g for 3 minutes at 4 degrees. Supernatant was discarded and pellets were resuspended with 2 mL of citric acid-based buffer (sucrose 0.25 M, citric acid 25 mM, $MgCl_2$ 3mM) then pass through a 35 μm cell strainer. The flow-through was centrifuged at 800 g for 3 minutes at 4 degrees. The supernatant was discarded and pellets were resuspended with 0.5 mL of storage buffer (KCl 25 mM, $MgCl_2$ 3mM, Tris 25 mM, SuperaseIn 1 U/μL, cOmplete protease inhibitor (4693159001, sigma), 40% Glycerol). Nuclei in storage buffer were transferred to 1.5 mL microcentrifuge tubes and froze at −80 degrees.

Frozen nuclei pellets were thawed in 50 μL 10% PBS-BSA, pelleted (800 g, 3 min, 4°C), and washed with 1 mL 2% PBS-BSA. Nuclei were counted, pelleted again, and resuspended at 5x10^6 nuclei/mL in cold PBS. ~200k nuclei were aliquoted from each sample for labeling with MULTI-seq barcodes (McGinnis et al., 2019). After labeling, nuclei were washed once with 1 mL 2% PBS-BSA, pelleted (600 g, 4 min, 4°C), resuspended in 200μL 2% PBS-BSA, and then pooled. The pooled sample was counted, and cDNA libraries were generated using two lanes of a 10X Genomics Chromium Next GEM Single Cell 3′ v3.1 reagent kit, targeting 10k nuclei per lane. MULTI-seq barcode libraries were generated according to the MULTI-seq protocol (https://github.com/chris-mcginnis-ucsf/MULTI-seq). The quality of all libraries was assessed using the High Sensitivity DNA kit for the Agilent 2100 Bioanalyzer. cDNA and barcode libraries were multiplexed and submitted to the UCSF Center for Advanced Technology for sequencing on a NovaSeq 6000 SP flow cell, yielding 518M reads.

cDNA reads from both libraries were aligned to the GRCh38-3.0.0 reference transcriptome with Cell Ranger v5.0 (https://support.10xgenomics.com/single-cell-gene-expression/software/downloads/latest), using the option "–include-introns" to enable detection of unspliced mRNAs. Using the Seurat v3.2 R package (Stuart et al., 2019), filtered gene expression matrices from each library were loaded and then integrated into a single merged dataset. MULTI-seq barcode libraries were loaded and parsed with the deMULTIplex v1.0 R package (https://github.com/chris-mcginnis-ucsf/MULTI-seq) to assign each nucleus to its sample of origin. Doublets, unclassifiable nuclei, and samples with insufficient nuclei recovery were removed from the dataset, yielding a final dataset of 4,450 nuclei. From these we captured an average of 959 UMIs and 780 genes per nucleus. Mitochondrial genes were removed from the dataset to mitigate biases arising from differences in nuclei isolation between samples. Expression data were normalized with SCTransform (Hafemeister and Satija, 2019). The first 32 principal components were used for UMAP embeddings and to calculate the k nearest neighbors per cell, followed by cluster identification using the Louvain algorithm.

Using the sample classifications derived from MULTI-seq barcodes, Clusters 1 & 3 were identified as Pancreatic Progenitor nuclei, Cluster 2 as Day 8 Ductal Organoid nuclei, and Clusters 0 and 4 as Day 8 Acinar Organoid nuclei. Using these new groupings, marker genes were calculated using FindAllMarkers with a logFC threshold of 0, and only those with an adjusted p value < 0.05 were kept for downstream analysis. The marker heatmap was produced using Seurat's DoHeatmap function by downsampling all three cell types to the same number and using only marker genes overexpressed by 20%, i.e., avg_logFC > log(1.2). Pearson correlation coefficients were calculated pairwise between acinar organoids, ductal organoids, and progenitors using the average expression of all genes in the dataset computed by Seurat's AverageExpression function.

As a reference point against which to compare our organoids, single-nucleus RNA-seq data of the human neonatal pancreas was downloaded from http://singlecell.charite.de/pancreas/ (Tosti et al., 2021) and filtered to contain only the acinar and ductal cells.

Using markers derived from organoids and progenitors (adjusted p value $\leq$ 0.05), we calculated average expression profiles for each cell type in both datasets and computed Pearson correlation coefficients pairwise between them.

QUANTIFICATION AND STATISTICAL ANALYSIS

For data regarding RNA expression quantitated by qPCR, enzyme activities, protein marker expression detected by immunofluorescent staining, Alcian blue staining, cell number changes, percentages of organoids with lumen changes, t test was used for testing the statistical significance and $p = 0.05$ was used as the threshold. For data regarding organoid size and form factor changes, Ki67 expression, $p16^{INK4A}$ expression, Mann-Whitney test was used for testing the statistical significance and $p = 0.05$ was used as the threshold. Details for chart representation and experimental replication can found in figure legend.

Single nuclei sequencing results were analyzed using the Seurat package in R language. Statistical significance of differences in gene expression was tested using the Wilcoxon Rank Sum test and $p = 0.05$ was used as the threshold. Details can be found in experimental procedures.

Cell Stem Cell, Volume *28*

Supplemental Information

Commitment and oncogene-induced plasticity of human stem cell-derived pancreatic acinar and ductal organoids

Ling Huang, Ridhdhi Desai, Daniel N. Conrad, Nayara C. Leite, Dipikaa Akshinthala, Christine Maria Lim, Raul Gonzalez, Lakshmi B. Muthuswamy, Zev Gartner, and Senthil K. Muthuswamy

A

	iPS11	iPS13
Donor Age	26	27
Donor Gender	Male	Female
Donor weight (lbs)	215	106
Donor BMI (CDC calculator)	32.7	21.4
Donor Race		
Health Information	Healthy	Healthy
Tissues used for generating iPS cells	Eritroblasts	Eritroblasts
Passages used for experiments	9	18

B

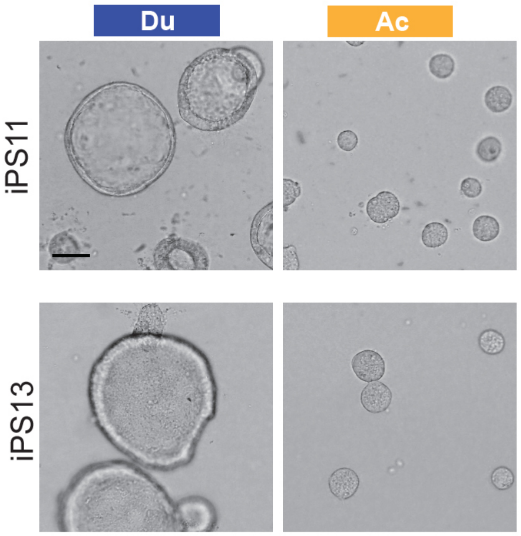

C

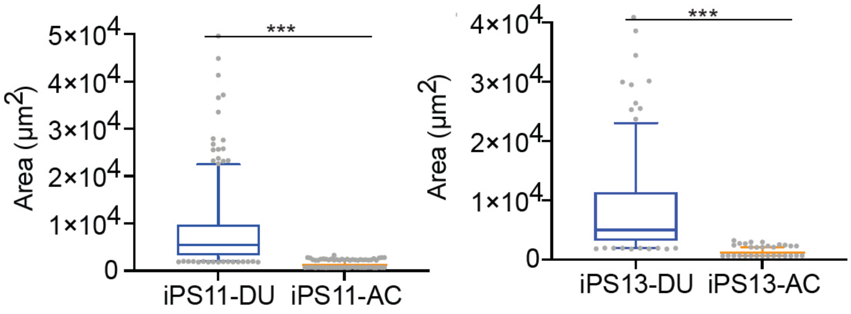

Figure S1. Induction of duct-like and acini-like organoids using progenitors derived from iPS cells (related to Figure 1) **(A)** Donor information for iPS cell lines used. **(B)** Phase contrast images of duct-like (DU, blue) and acini-like (AC, orange) organoids using iPSC-derived pancreatic progenitors. Scale bard, 50 µm. **(C)** Quantification of organoid sizes (N = minimal 200). Whisker-box plot, range 5-95%; center lines indicate median values; grey dots represent individual measurements. ***, $p<0.001$. Results were summary from three independent cultures.

A

	Gene Expression (arbitary unit, x10-3)		
	PP	DU	AC
PDX1	29.72	78.85	106.52
SOX9	25.97	229.48	4.66
HNF1B	141.21	175.75	84.28
HNF1A	21.85	57.07	15.53
RBPJL	4.12	12.68	12.20
RBPJ	630.67	1742.17	639.18
CPA2	2.62	6.34	4.66
CEL	6.12	6.34	15.31
PNLIP	1.75	0.00	18.42
CTRB1	0.00	0.00	3.88
CTRC	2.62	0.00	4.66

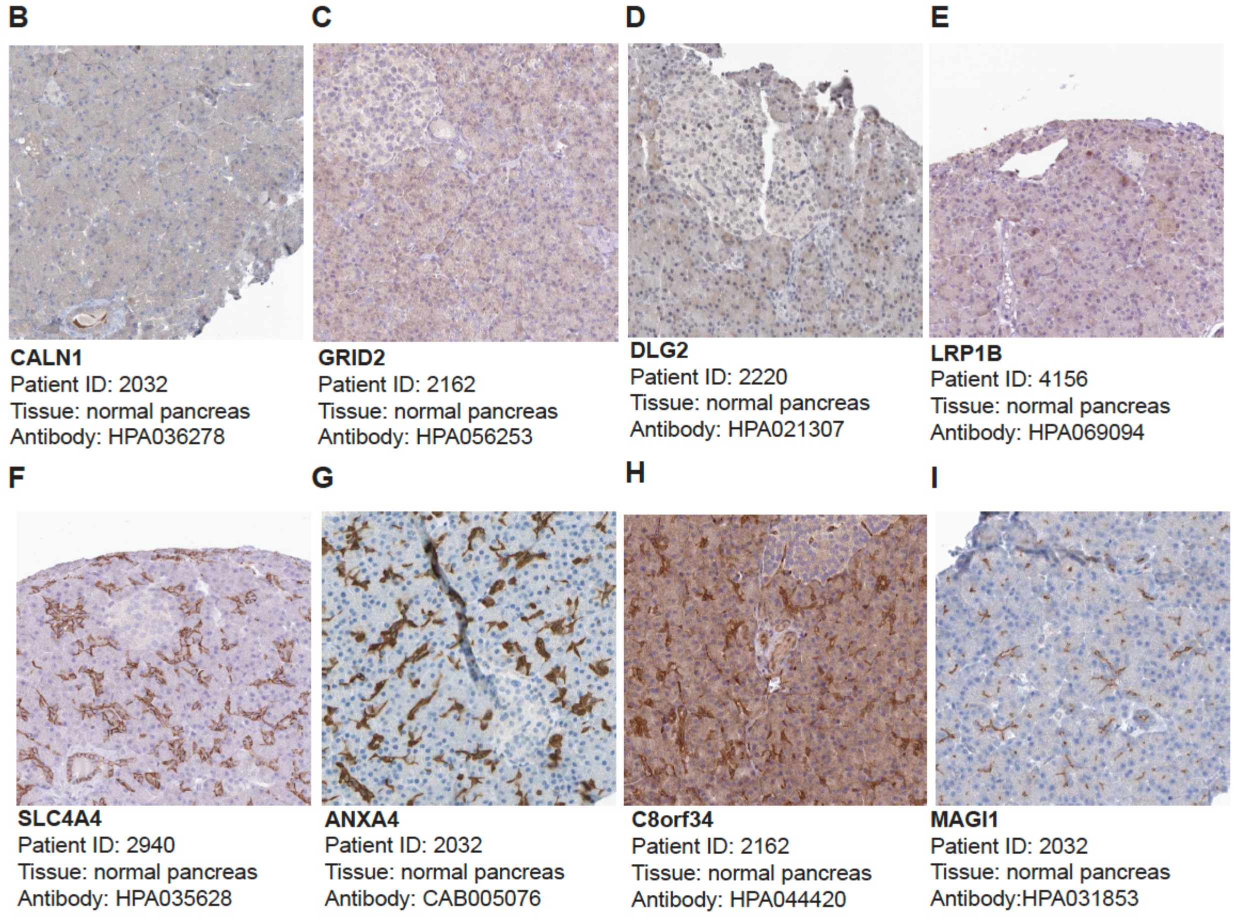

Figure S2. Single nuclei RNA sequencing detects expression of pancreatic differentiation markers in organoids (related to Figure 2) **(A)** Expression of classical pancreatic lineage markers in different cell groups. Results were obtained from two independent organoid cultures and separate sequencing. **(B-E)** Expression of acini-like organoid-enriched markers in normal human pancreas. Images obtained from Human Protein Atlas. **(F-I)** Expression of duct-like organoid-enriched markers in normal human pancreas. Images obtained from Human Protein Atlas.

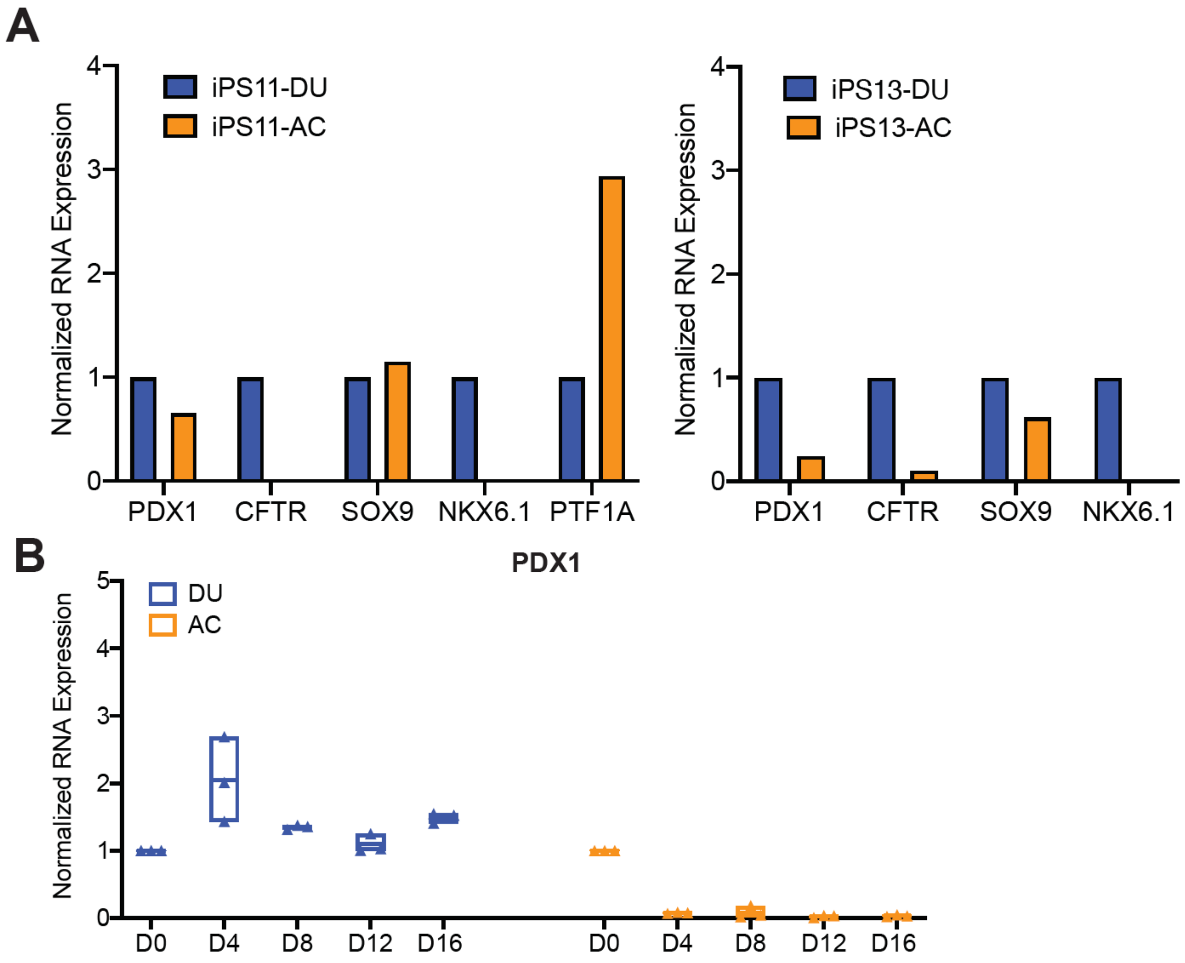

Figure S3. Expression of Pancreatic lineage markers during organoid morphogenesis (related to Figure 3) **(A)** Pancreatic lineage marker expression in organoids induced from iPSC-derived pancreatic progenitors. Results from one batch of organoid culture. **(B)** *PDX1* RNA levels were quantitated by quantitative PCR. Floating column charts represent RNA measurements from quantitative PCR (N=3, independent cultures); Hinges represent maximal and minimal values, central lines indicate mean values; dots represent individual measurements.

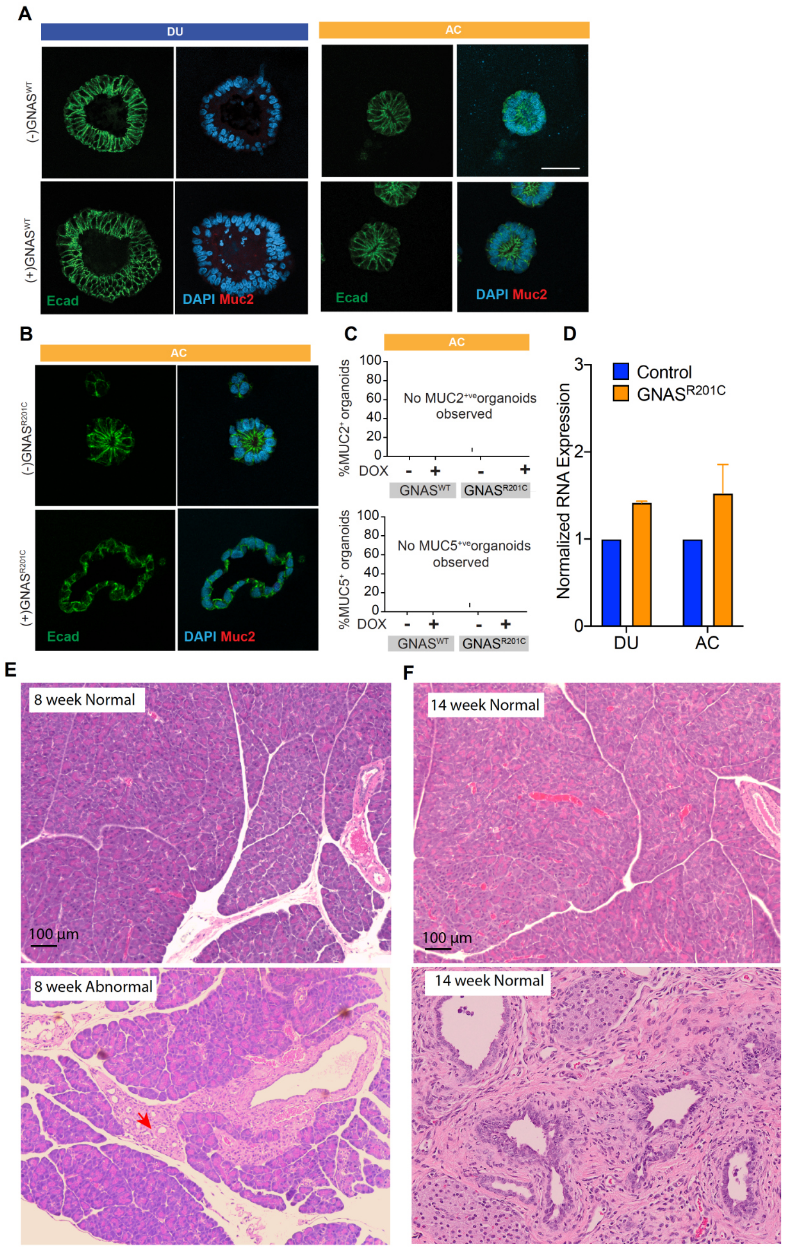

Figure S4. Effects of wildtype and mutant GNAS on organoid differentiation (related to Figure 4) **(A)** Expression of MUC2 and E-cadherin in duct-like and acini-like organoids with and without expressing wildtype GNAS (N=3, independent cultures). MUC2, red; E-cadherin, green; DAPI, blue. Scale bar, 100 µm. **(B)** Expression of MUC2 and E-cadherin in acini-like organoids with and without expressing $GNAS^{R201C}$ (N=3). MUC2, red; E-cadherin, green; DAPI, blue. Scale bar, 100 µm. **(C)** Quantification of MUC2 and MUC5AC expression in acini-like organoids with and without expressing $GNAS^{R201C}$ (N=3). **(D)** CFTR RNA expression in organoids with $GNAS^{R201C}$. N= 2, biological repeats. **(E-F)** Normal (top panels) and abnormal (bottom panels) mouse pancreatic tissues with (hematoxylin and eosin stained) at eight weeks (D) (N=8) and 14 weeks (E) (N=4) post transplantation with duct-like organoids expressing $GNAS^{R201C}$. Scale bars, 100 µm.

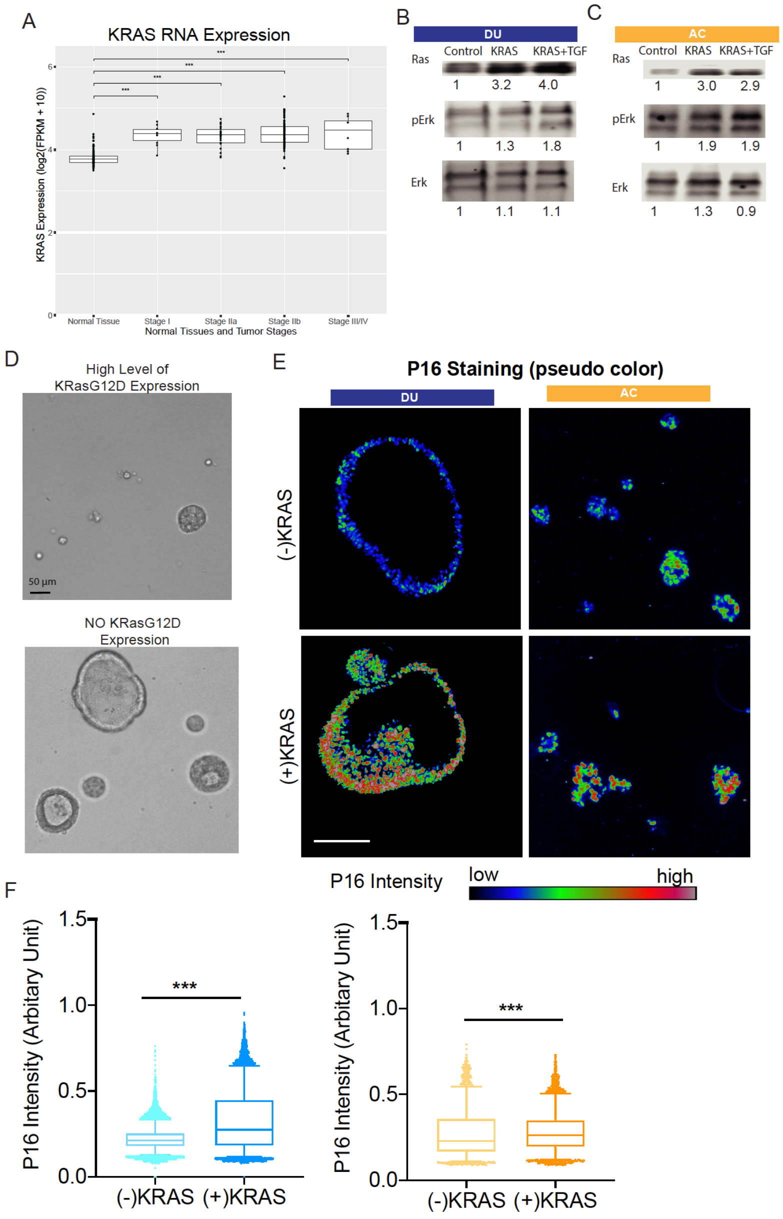

Figure S5. Effects of *KRAS*^{G12D} expression on organoids (related to Figure 5) (A) Expression of *KRAS* in the pancreas of healthy subjects and PDAC patients. Data obtained from The Cancer Genome Atlas. **(B-C)** Immunoblot analysis for KRAS and ERK phosphorylation in duct-like (B) and acini-like (C) organoids. Numbers blots represent normalized intensities of protein bands. **(D)** Phase contrast images of duct-like organoids expressing high levels of *KRAS*^{G12D} (top panel) and organoids without *KRAS*^{G12D} expression (bottom panel). **(E)** Pseudocolor images indicating p16^{INK4A} intensities in organoids without (top panel) and with (bottom panel) *KRAS*^{G12D} expression. Images converted from immunofluorescent staining of p16^{INK4A}. Scale bars, 100 µm. (**(F)** Quantification of p16^{INK4A} expression per nuclei in organoids without and with *KRAS*^{G12D} expression (N>2500, from three independent cultures). Y-axis present signals normalized to maximal values of 8 bit image (255). Whisker-box plot, range 5-95%; center lines indicate median values; grey dots represent individual measurements. ***, p<0.001.

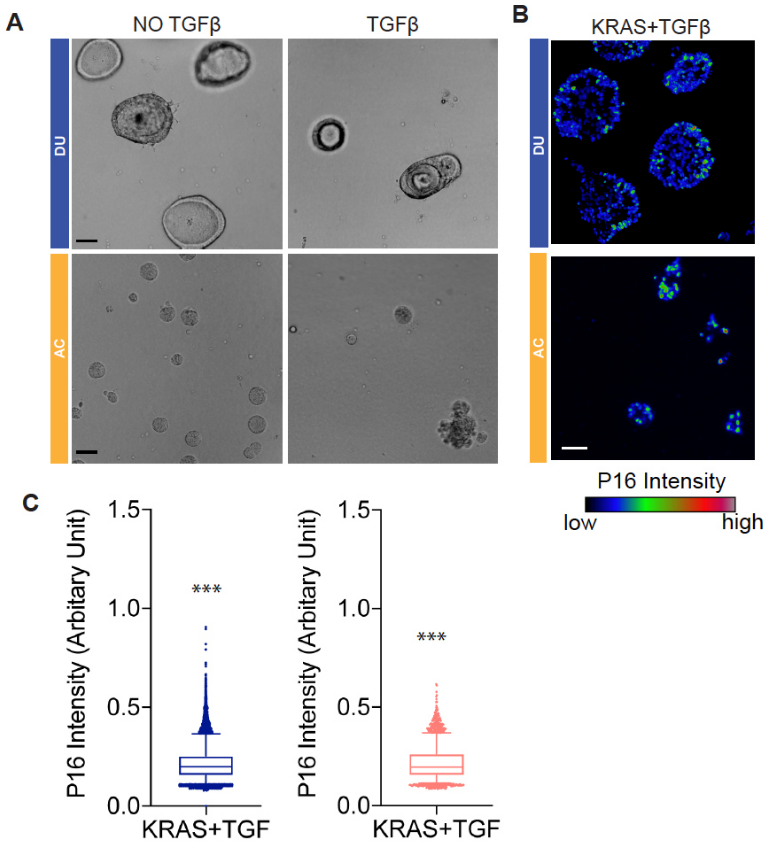

Figure S6. TGFβ treatments induce biological changes in organoids (related to Figure 6) **(A)** Phase contrast images of duct-like and acini-like organoids without and with TGFβ treatments. Scale bar, 50 µm. (**B)** Pseudocolor images indicating p16^{INK4A} intensities in *KRAS*^{G12D} expressing organoids with TGFβ treatments. Images converted from immunofluorescent staining of p16^{INK4A}. Scale bars, 100 µm. **(C)** Quantification of p16^{INK4A} expression per nuclei in *KRAS*^{G12D} organoids with TGFβ treatments (N>2500, from three independent cultures). Y-axis present signals normalized to maximal values of 8 bit image (255). Whisker-box plot, range 5-95%; center lines indicate median values; grey dots represent individual measurements. ***, p<0.001.

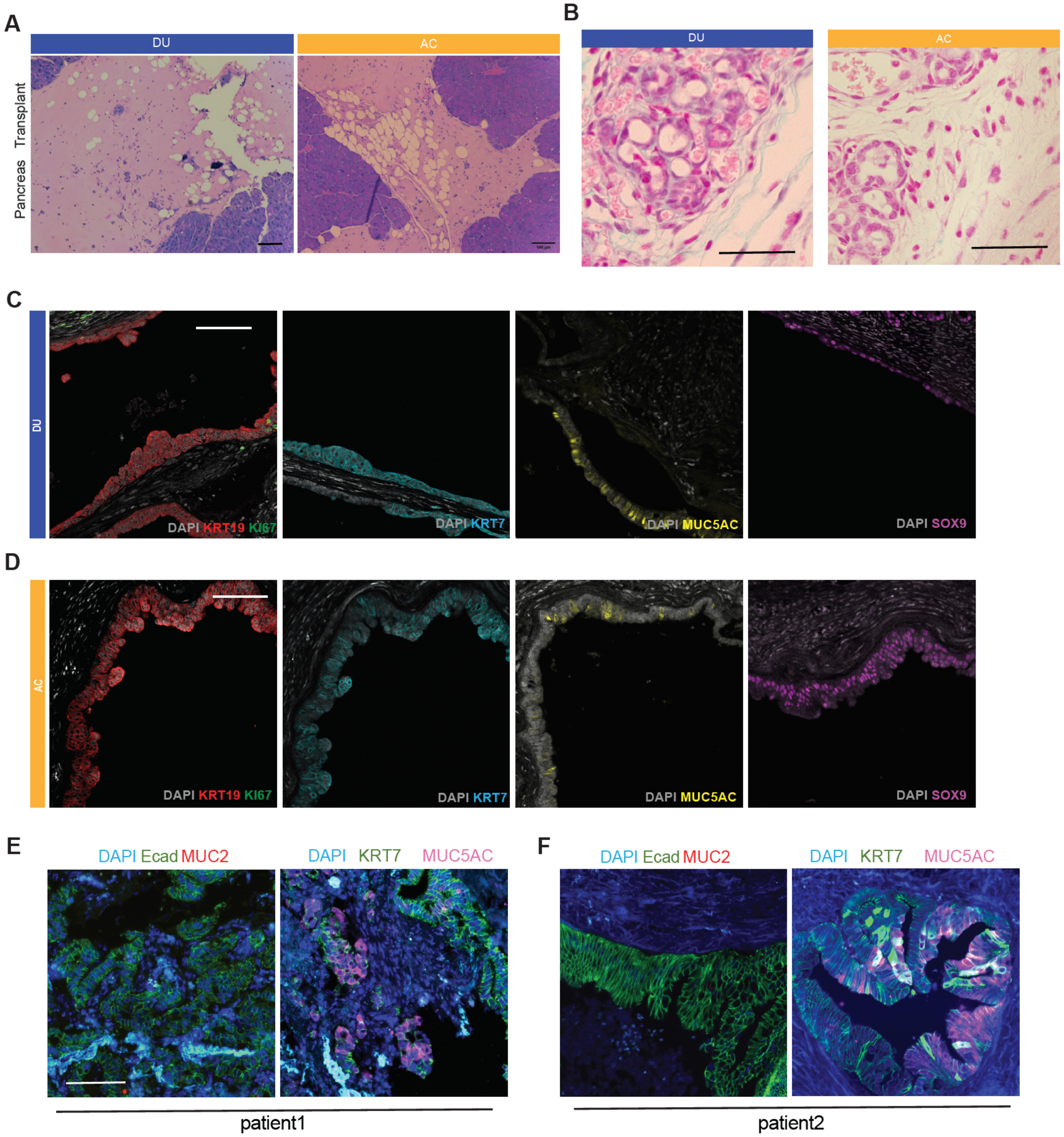

Figure S7. Orthotopic transplantation of organoids into mice (related to Figure 7) (A) H&E images of mouse pancreas transplanted with duct-like (DU) and acini-like organoids (AC) without oncogene expression (N=10 mice for each group). Scale bars, 100 µm. **(B)** Mouse pancreatic tissues adjacent to organoid-derived lesions exhibiting pancreatitis-like histology. Scale bars, 100 µm. **(C-D)** Protein expression in lesions grown from *KRAS*^{G12D} expressing duct-like (C) (N=9 mice) and acini-like (D) (N=10 mice) organoids. KRT19, red; Ki67, green; KRT7, teal; MUC5AC, yellow; SOX9, purple. Scale bars, 100 µm. **(E-F)** Protein expression in human PDAC tumor tissues. Left panel: E-cadherin, green; MUC2, red; DPAI, blue. Right panel: KRT7, green; MUC5AC, red.
